# Integrated analysis of shared gene expression signatures and immune microenvironment heterogeneity in type 2 diabetes mellitus and colorectal cancer

**DOI:** 10.1038/s41598-025-07015-4

**Published:** 2025-07-01

**Authors:** Zhaohui Wu, Liuliu Cao, Jie Zhao

**Affiliations:** 1 Digestive System Department, Second Hospital of Shanxi Medical University, Taiyuan, Shanxi China; 2 Shanxi Medical University, Taiyuan, Shanxi China; 3 Department of Emergency, The Affiliated Hospital of North China University of Science and Technology, Tangshan, Hebei China; 4 North China University of Science and Technology, Tangshan, Hebei China

**Keywords:** Colorectal cancer, Type 2 diabetes mellitus, Machine learning, Single-cell, Biological techniques, Cancer, Computational biology and bioinformatics, Gastroenterology, Medical research, Oncology

## Abstract

Emerging evidence suggests a bidirectional relationship between colorectal cancer (CRC) and type 2 diabetes mellitus (T2DM), yet the shared molecular mechanisms and prognostic biomarkers remain poorly characterized. This study aimed to identify novel biomarkers linking CRC and T2DM pathogenesis and evaluate their clinical utility in predicting therapeutic responses and survival outcomes. By integrating multi-omics data from public repositories and applying machine learning-driven feature selection, we identified three core biomarkers—*FABP4*,*CDR2L*,and *FSTL3* that independently predicted overall survival in CRC patients with diabetes. A prognostic nomogram combining these biomarkers with clinicopathological variables (tumor stage, grade, and age) achieved high accuracy for 1-, 3-, and 5-year survival prediction. Functional characterization revealed strong associations between biomarker overexpression and tumor microenvironment remodeling, particularly through fibroblast-mediated immune cell recruitment and cross-talk with lymphocytes. Critically, low expression of these genes correlated with improved anti-PD-1 immunotherapy responses in an independent clinical cohort. Our findings establish *FABP4*, *CDR2L*, and *FSTL3* as pivotal regulators at the CRC-diabetes interface, with dual utility as prognostic indicators and predictors of immunotherapy efficacy.

**Supplementary Information:**

The online version contains supplementary material available at 10.1038/s41598-025-07015-4.

## Introduction

Globally, colorectal cancer (CRC), one of the most prevalent malignant tumors, has approximately 1.92 million new cases and 900,000 deaths annually, accounting for around 10% of all cancers^1^. It is widely acknowledged that the immune microenvironment where colorectal cancer tumors reside plays a crucial regulatory role. The progression of colorectal cancer includes multiple oncogene mutations, such as *KRAS* and *TP53*. These mutations prompt the release of specific signaling molecules and cytokines, subsequently altering the tumor microenvironment^2^. Given the pivotal role of the immune system within the tumor microenvironment, immunotherapy targeting the immune system has emerged as a treatment option for colorectal cancer. To gain a better understanding of CRC’s response to immunotherapy, identify new immunotherapy targets, and consequently develop novel and effective treatment strategies is urgently needed.

In 2021, there were 529 million diabetic patients worldwide, with an age-standardized prevalence of 6.1%^3^. Among them, type 2 diabetes accounts for over 90%. With the intensifying trend of population aging in the future, the number of patients with type 2 diabetes mellitus (T2DM) will continue to rise. According to existing epidemiological surveys, type 2 diabetes, recognized as a systemic chronic inflammatory disease, is closely associated with the incidence of malignant tumors. In patients with CRC, risk factors like high-calorie intake, high-fat diet, and overweight, which are also prevalent in T2DM patients, suggest a certain correlation between the occurrence and development of the two^4^. T2DM leads to systemic inflammation resulting in the release of a large number of inflammatory factors, and the inflammatory pathways such as *TGF-β* and *IL-6*, which are shared with CRC, can interfere with the normal activation signaling pathways of immune cells, and are unable to efficiently play the role of immune defense and clearance^5,6^. It has been shown that hyperglycemia enhances tumor glycolysis and inhibits CD8 + T cell function through *HIF-1α*^7^. In addition, T2DM therapeutic agents (e.g., metformin) may regulate flora and inhibit CRC through the *AMPK* pathway, but their immunometabolic disorders may also affect anti-PD-1 efficacy^8,9^.

Although there is compelling evidence in clinical and epidemiological fields demonstrating the interaction between type 2 diabetes and colorectal cancer, the common mechanism of action and key molecular characteristics involved in gene regulation remain largely unclear. It is of great significance to elucidate the pathogenic mechanism and molecular markers of the reciprocal crosstalk between type 2 diabetes and colorectal cancer to enhance disease diagnosis, treatment, and prognosis strategies.

## Methods

### Selection of gene expression profiles for T2DM and CRC

In the present study, we systematically retrieved T2DM data (GSE118139, GSE164416, GSE184050, GSE25724, GSE38642, GSE50397, GSE50244) and CRC data (GSE9348, GSE113513, GSE17536, GSE17537) as well as single cell data (GSE200997) from the Gene Expression Omnibus (GEO) database. Concurrently, the TCGA-COAD cohort in the Cancer Genome Atlas (TCGA) database was also chosen, and the R package tcgabiolinks was employed to acquire the counts per million (CPM) of gene expression fors ubsequent analysis^10^. The summary statistics of all datasets are presented in Table S1.

### Identification of differentially expressed genes (DEGs)

Firstly, the limma package was utilized to normalize T2DM data (GSE118139), CRC data (GSE9348), and the TCGA-COAD cohort. To identify the key co-expressed molecules related to the diseases, the “ebayes” method within the limma package was employed to optimize the variance estimation during differential expression analysis^11^. Additionally, the standard error was adjusted to more precisely estimate the significance of gene expression differences, thereby determining the significantly differentially expressed genes in T2DM and CRC. *P* < 0.05 and logFC ≥ 1.

### Functional annotation and gene set enrichment analysis

The protein-protein interaction (*PPI*) network disclosed both specific and non-specific interactions between proteins, and it is also capable of identifying core protein-coding genes. The STRING database serves as a commonly utilized resource for retrieving known proteins and predicting relationships between core protein genes. To explore the potential biological processes associated with the obtained differentially expressed genes, the ‘clusterProfiler’ R package was employed for enrichment analysis^12^. Specifically, two major were involved: First, the Kyoto Encyclopedia of Genes and Genomes (KEGG), Many biological pathways containing various organisms^13–15^; Second, the Gene Ontology (GO), which was analyzed from three aspects of biological processes, namely cellular components (CC), molecular functions (MF), and biological processes (BP)^16^. Relevant gene sets were downloaded from the MSigDB database (https://www.gsea-msigdb.org/gsea/msigdb). After converting the IDs via the “org.hs.egg.db” R package, the “enrichGO” and “enrichKEGG” functions within the “clusterProfiler” R package were utilized to analyze biological processes and key pathways.

### **Machine learning model strategies**

The LASSO-Cox regression model was initially applied for preliminary risk stratification using differentially expressed genes (DEGs), which were subsequently used as input features for machine learning algorithms to identify critical prognostic biomarkers.Input features comprised normalized expression levels of candidate DEGs, with binary classification (high-risk vs. low-risk) serving as the output variable. The dataset was partitioned into training and validation subsets at a 7:3 ratio. The caret package was employed for model training, utilizing three repetitions of 5-fold cross-validation to control resampling procedures during the training phase. The main parameters of each of these models are summarized below: Random Forest(RF): 500 trees, maximum depth = 3, mtry = 5; Gradient Boosting Machine(GBM): n.trees = 100, interaction depth = 1, learning rate = 0.1; Decision Tree(DT): Maximum depth = 5; LASSO regression (α = 0.01) emphasized feature sparsity through L1 regularization, with optimal lambda selection via cv.glmnet; Generalized Linear Model(GLM) utilized L2 regularization (binomial family) to preserve baseline interpretability; The neural network(NNET) architecture incorporated: A single hidden layer neuron to constrain complexity, weight decay = 0, Training limitation to 1000 iterations; K-Nearest Neighbors (kNN): the number of neighbors was set to k = 2; The Support Vector Machine (SVM) utilized the Radial Basis Function (RBF) kernel with a regularization parameter set to a balanced default value (C = 1.0), implemented via the svmRadial package in R^17–19^.Model performance evaluation using the DALEXR package.

### Construction of prognostic risk models

Firstly, survival objects were established based on patient survival status and gene expression data. Subsequently, risk models were constructed through the log-rank test, univariate and multivariate Cox regression analyses. During this process, the set of genes with significant risk ratios, along with other relevant clinical data, was extracted. Binary inputs were employed, which were determined by whether the gene abundance was above or below the median. To prevent the coefficients of certain unimportant features from being involved in prognostic risk prediction, we utilized the Least Absolute Shrinkage and Selection Operator (LASSO) regression to reduce dimensionality and select the most reliable markers for gene evaluation^20^. The optimal value of the penalty parameter λ was ascertained via 10-fold cross-validation^21^. Finally, clinical predictive modeling was carried out using stepwise regression logic and nomogram.

### Multi-omics analysis of identified key molecules

The Human Protein Atlas (HPA) (https://www.proteinatlas.org/) was utilized to identify the protein expression of immunohistochemically stained key genes in patients with T2DM and COAD. This aimed to further clarify the manifestation of pathological changes in both diseases at the protein level. Additionally, we identified the biological pathways activated or inactivated in the genes through gene-set enrichment analysis (GSEA) of expression data adjusted for CRC transcripts. The gene sets were obtained from the MSigDB database. The gmt file (c2.cp.kegg_medicus.v2024.1.Hs.entrez.gmt) was downloaded, and the “GSEA” function in the R package “clusterProfiler” was used to perform GSEA analysis^22^. *P*-value cutoff < 0.05.

### Assessment of immune cell infiltration

Based on Gabriela Bindea’s study^23^, single-sample gene-set enrichment analysis (ssGSEA) was employed to quantify the immune cell infiltration of each immune cell type in both the diabetes samples from the T2DM dataset (GSE164416) and the tumor samples from the TCGA-COAD cohort. Specifically, the fraction of each immune cell enriched in individual samples was calculated to infer the infiltration of immune cells in each sample.Meanwhile, to account for the bias introduced by tumor purity, according to Kosuke’s study^24^, the ESTIMATE algorithm was used to calculate the CRC tumor purity and obtain the StromalScore (indicating the level of stromal cell infiltration), ImmuneScore (indicating the level of immune cell infi ltration), and EstimateScore (indicating the level of non-tumor cell infiltration) of the samples. Moreover, the relationships between prognostic characteristics, immunoreactivity-related characteristics^25^, and immune checkpoint-related characteristics were analyzed using Spearman’s coefficient and Wilcoxon rank-sum test. In total, 28 human immune cell subtypes were evaluated.

### scRNA-seq analysis

Single-cell data from colon cancer were obtained from the GEO dataset (GSE200997) using the R Seurat software package (version 5.1.0). 10x genomics data from 23 patients were read to process the data for constructing Seurat objects. Low-quality cells were filtered according to the quality control criteria: cells with more than 200 genes and less than 6000 genes, and cells with a mitochondrial RNA percentage greater than 25% were excluded. These strict selection criteria and quality control steps were implemented to ensure the high quality and reliability of our data. Significant principal components (PCs) were identified via principal component analysis (PCA)^26^. The top 17 PCs were selected as the statistically significant inputs (dims = 17). Inter-image distances were calculated using the FindNeighbors function, which guided the construction of the shared nearest-neighbor graph. Cell clustering and cell-type identification of cell clusters were then analyzed using the ‘FindClusters’ function. For data visualization, cell data were projected using UMAP (Uniform Manifold Approximation and Projection). To identify feature genes, the FindAllMarkers function was applied, and significant genes were identified using the Wilcoxon rank-sum test. The marker genes identified from the feature set were then used to assign cell subpopulations, referring to the CellMarker database^27^ and related literature^28–33^.

### Intercellular interactions

Intercellular interactions are crucial for various biological processes^34^. A cell’s biological behavior is regulated by both its intracellular regulatory network and extracellular signaling environment, which ultimately determines the cell’s function. Intercellular pathway interactions were analyzed using CellChat, a web-based tool for assessing ligand-receptor interactions between cell types. The CellChatDB.human database was utilized to obtain relevant pathway information. Subsequently, the gene expression data was projected onto a *PPI* network. The ligand-receptor interaction analysis functions, computeCommunProb and computeCommunProbPathway, were used to calculate the probability of cell-to-cell communication and the probability of cellular communication for a specific signaling pathway, respectively. The “netVisual_aggregate” and “netVisual_individual” functions were used to visualize the inferred signaling pathway communication network^35,36^.

### Immunotherapy response prediction

Immunotherapy plays a crucial role in the current treatment of CRC. The IMvigor210 cohort consisted of patients with locally advanced or metastatic bladder cancer who underwent immunotherapeutic intervention with atezolizumab after multiple lines of therapy had failed. The cohort provided detailed baseline characteristics of the patients, encompassing information such as age, gender, tumor stage, and the treatment^37^. We downloaded complete transcriptomic data and detailed clinical information from (http://research-pub.Gene.com/imvigor210corebiologies). Normalization was carried out using the DEseq2 R package, and then the counts were converted to TPM values. Supplementary Table provides detailed information on the enrolled subjects for each dataset.Regarding efficacy assessment, tumor remission in patients was accurately determined according to the RECIST criteria^38^. This involved statistics on the proportions of complete remission (CR), partial remission (PR), stable disease (SD), and disease progression (DP). Subsequently, key indices such as the objective remission rate (ORR) and non-remission rate (NRR) were calculated to reveal the impact of immunotherapy on patients’ survival outcomes.

### Statistical analysis

Initially, all statistical analyses were conducted using R software (version 4.3.2).For two independent continuous variables, differences were evaluated using Student’s t-test or the Wilcoxon rank-sum test. A paired t-test was employed to assess the difference between two paired continuous variables. When dealing with three or more continuous variables, one-way ANOVA or the Kruskal-Wallis test was utilized for analysis. Pearson’s test or Spearman’s test was applied to assess the correlation between two variables. *P* < 0.05 as statistically significance.

## Results

### Identification of DEGs in diabetes and Colon cancer

**Fig. 1:**
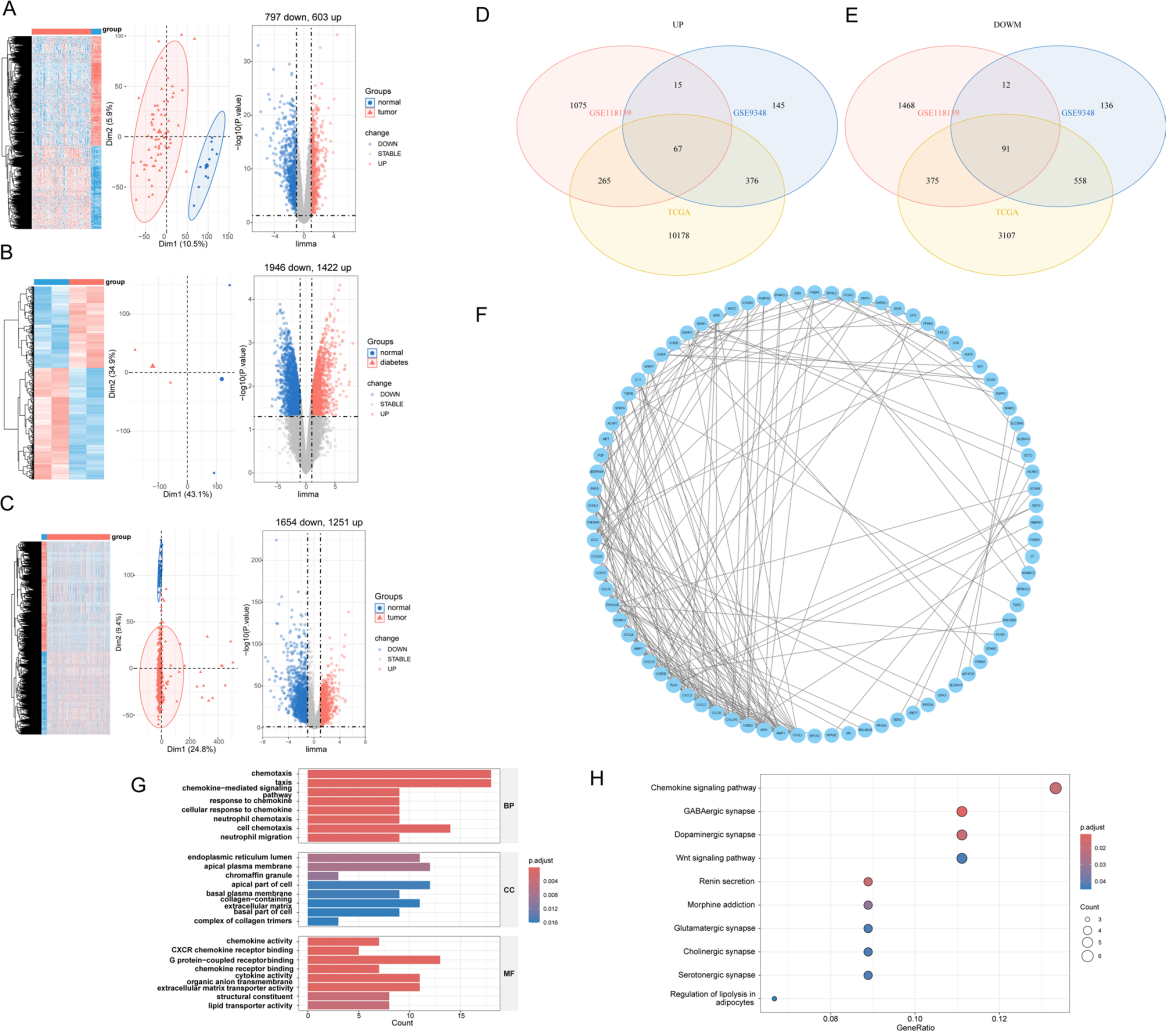
Acquisition and analysis of shared DEGs between T2DM and CRC. (**A**) Volcano plot, PCA, and Heatmap of GSE118139. (**B**) Volcano plot, PCA, and Heatmap of GSE9348. (**C**) Volcano plot, PCA, and Heatmap of TCGA-COAD. (**D**) Venn diagram of up-regulated DEGs between T2DM and CRC. (**E**) Venn diagram of down-regulated DEGs between T2DM and CRC. (**F**)PPI network of the shared DEGs. (**G, H**). (**G**) GO and (**H**) KEGG enrichment analysis of DEGs.

Overall, we utilized the GSE118139, GSE9348, and TCGA-COAD datasets. These datasets were normalized and processed through cluster analysis and principal component analysis (PCA). Subsequently, we identified DEGs, which were the differentially expressed genes. Heatmaps, PCA plots, and volcano plots were then used to visualize the up-and down-regulated differential expression in each tissue type (Fig. 1A–C).Through the intersection of Venn diagrams (Fig. 1D, E), we identified 158 DEGs with the same expression trend. Among them, 67 genes were up-regulated and 91 were down-regulated, with significant expression differences.

### PPI network and enrichment analysis

The identified differentially expressed genes (DEGs) were submitted to the STRING database to construct protein-protein interactions. Subsequently, the Cytoscape program (version 3.9.1) was used to construct *PPI* networks of the DEGs. Furthermore, key genes for analysis were identified using the Betweenness algorithm in the CytoNCA plugin to clarify the interaction relationships among the shared DEGs (Fig. 1F). In order to systematically assess whether DEGs are significantly enriched for immune-related biological processes, molecular functions or signaling pathways, GO and KEGG enrichment analyses were conducted to explore the biological processes associated with the initially screened differential genes. The enriched GO analysis revealed their involvement in biological processes such as cell growth, exogenous apoptosis signaling pathway, chemokine-mediated signaling pathway, G-protein-coupled receptor binding, cytokine activity, and chemokine receptor-binding chemokine signaling pathway (Fig. 1G). The enriched KEGG pathways were mainly associated with the regulation of the chemokine pathway, Wnt signaling pathway, glutamatergic synapses, and lipolysis in adipocytes (Fig. 1H). These potential biological functions were found to be significantly enriched in various metabolic, immune-related, and cancer-related pathways.

### Machine learning models construction

**Fig. 2:**
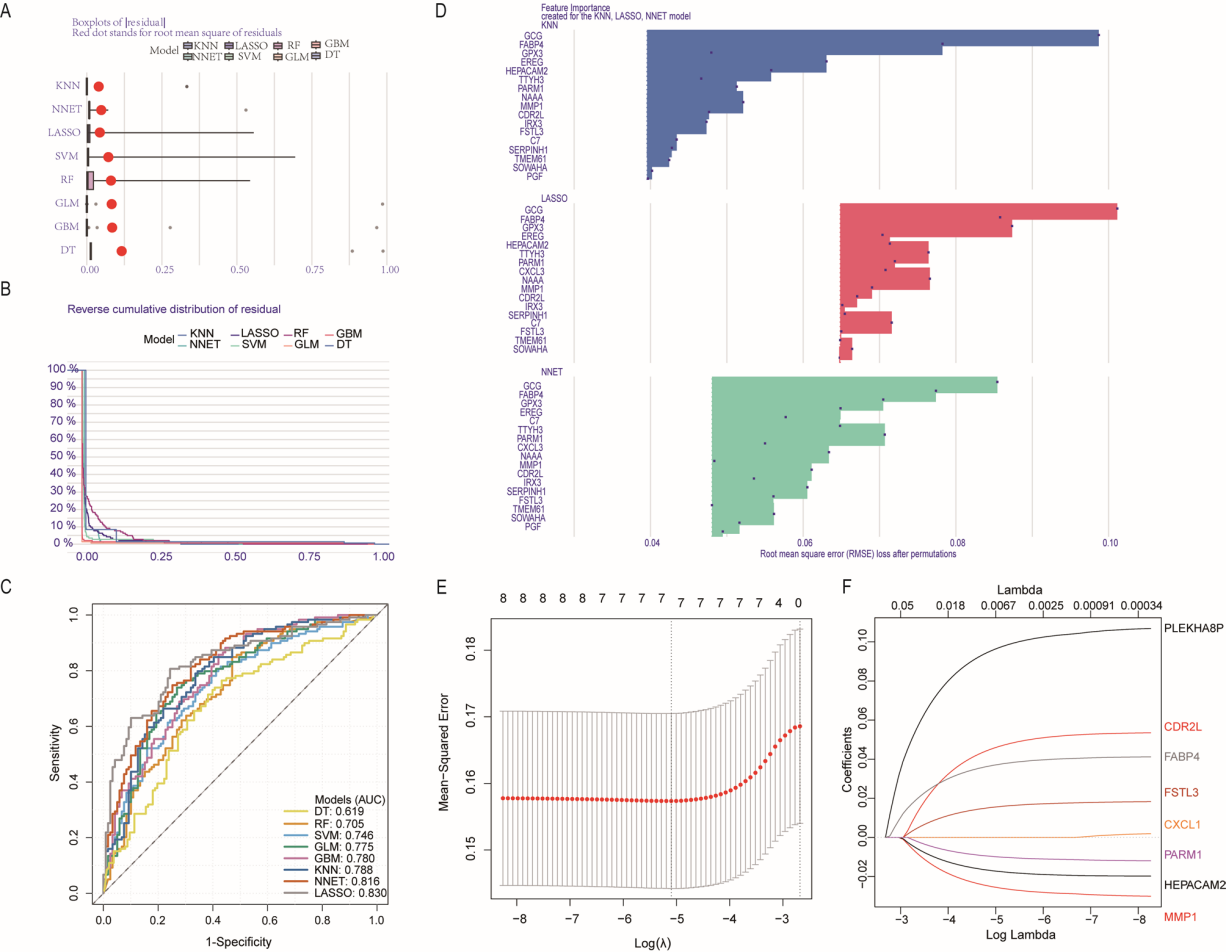
Screening of key genes. (**A, B**) Residual diagnostics analysis for key molecules among machine learning algorithm models, (**A**)the box plot of absolute residual distribution across prediction models; (**B**)the the reverse cumulative of the absolute residual plot. The red dot represented mean absolute residual; Residual is defined as the difference between actual value and the prediction. (**C**) The area under the curve (AUC) for the eight ML models. (**D**)Analysis and visualization of the feature importance created for the KNN model, LASSO model and NNET model. (**E**) Distribution map of least absolute shrinkage and selection operator (lasso) coefficients of key molecules. (**F**) Selection of the optimal parameter (lambda) in the lasso model. The dotted vertical lines showing the optimal values through minimum criteria and 1-s.e. criteria.

To assess genes related to survival and prognosis, survival analysis was conducted using the log-rank test (Table S2). Genes were quantified in univariate Cox regression analysis, and those with a *p* < 0.05 were selected (Table S3). This process was used to screen for the set of genes that were significantly correlated in both analyses. In order to identify the final set of core genes, we conducted a series of machine learning algorithm screenings. We calculated feature importance using the DALEX package, which provides an interpretable and reliable framework for understanding model predictions. The KNN model, Lasso model and NNET model has the lower median absolute residual value (Residual = True value - Predicted value) (Fig. 2A). From the reverse cumulative of the absolute residual plot, we can see that there is a higher number of residuals in the left tail of the GLM residual distribution (Fig. 2B). The LASSO model has the best AUC performance (AUC: 0.830), followed by NNET (AUC: 0.816), KNN (AUC: 0.788), GBM (AUC: 0.780), GLM (AUC: 0.775), SVM (AUC: 0.746), RF (AUC: 0.705), DT (AUC: 0.619): 0.619) had the worst performance(Fig. 2C). The genes with the higher importance scores were then selected for further analysis (Fig. 2D). Subsequently, the resulting set of genes was employed to establish a gene signature through the LASSO regression model(Fig. 2E,F).

### Multiple methods of key molecules

**Fig. 3:**
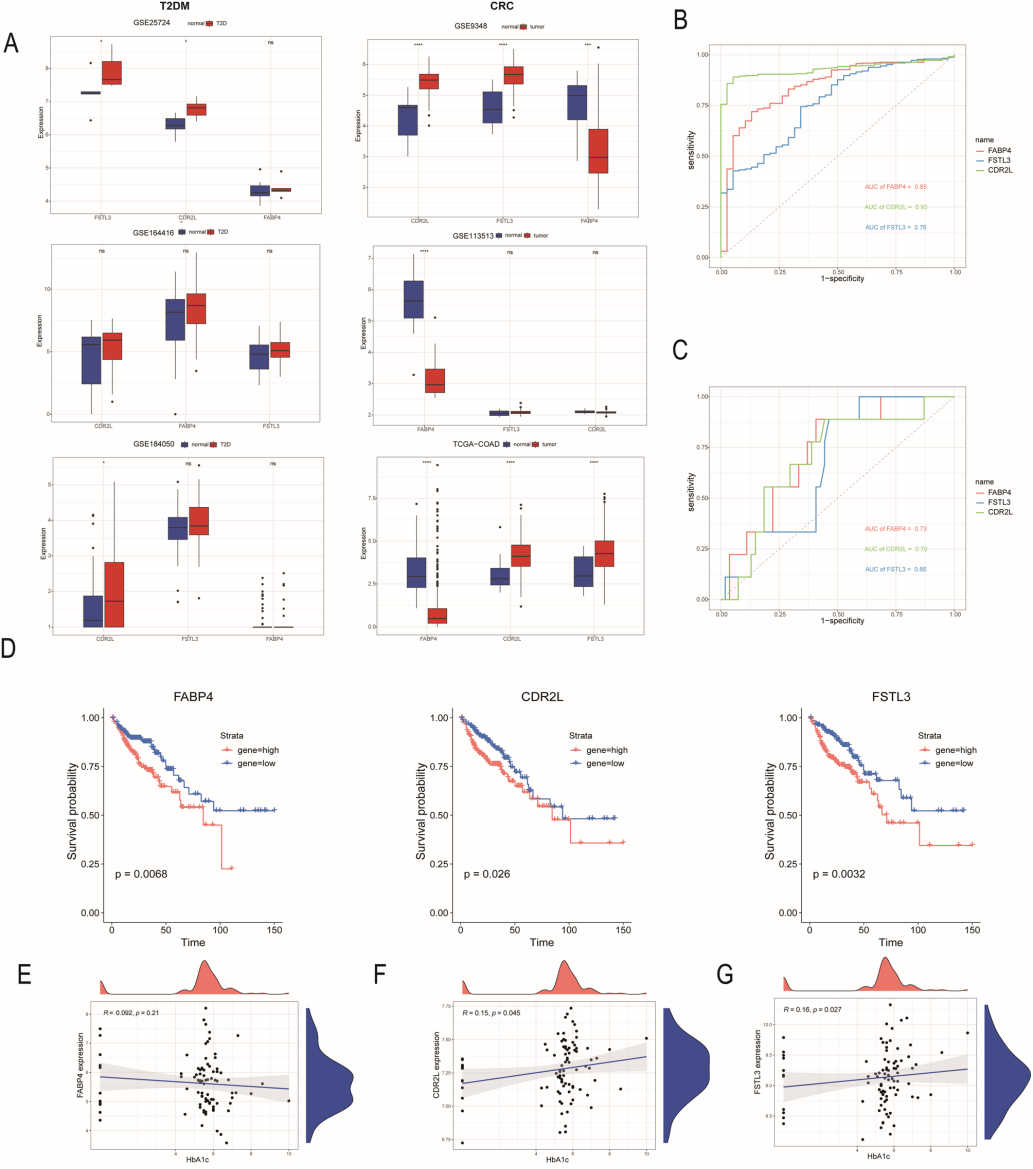
The expression, diagnostic, and prognostic value of FABP4, CDR2L, and FSTL3 in T2DM and CRC patients. (**A**) The expression of FABP4, CDR2L, and FSTL3 of T2DM and CRC. (**B, C**) ROC curve of FABP4, CDR2L, and FSTL3 in the (**B**) GSE38642 dataset and the (**C**) TCGA-COAD cohort. (**D**) K–M curve of association of FABP4, CDR2L, FSTL3 and CRC patients’ OS in the TCGA-COAD cohort. (**E-G**) Scatterplot of (**E**) CDR2L, (**F**) FSTL3 and (**G**) FABP4 expression and HbA1c level in the GSE38642 dataset. *p < 0.05, **p < 0.01, ***p < 0.001 and ns: *p *> 0.05.

The genes were further screened and optimized to prevent overfitting.Using a risk ratio (HR) > 1 as a key indicator, we identified three genes with high research potential and clear biological significance: fatty acid binding protein 4 (*FABP4*), cerebellar degeneration-related protein 2-like protein (*CDR2L*), and follicular stromelysin-like protein 3 (*FSTL3*). The expression levels of *CDR2L* and *FSTL3* were upregulated in both T2DM and CRC patient groups. Notably, *FABP4* exhibited an upregulated trend in the T2DM patient group, while a downward trend was observed in the CRC patient group(Fig. 3A). The ROC curves further demonstrated the diagnostic value of these key genes in the two diseases. The genes were validated using the GSE38642 (T2DM) and TCGA-COAD datasets. The area under the curve (AUC) values for *FABP4*, *CDR2L*, and *FSTL3* in T2DM were 0.73, 0.70, and 0.66, respectively, while in CRC, the AUC values were 0.85, 0.93, and 0.76, respectively (Fig. 3B,C).Survival curves illustrated the prognosis of patients. A total of 439 CRC patients with completed clinical annotation were included in the survival analysis., We found that patients with low expression of the three aforementioned key genes had a significant survival advantage compared to those with high expression (Fig. 3D).Glycated hemoglobin (*HbA1c*), an important indicator for assessing diabetes control, reflects not only the prognosis of diabetic patients but also the severity of diabetes. We found that *HbA1c* levels in T2DM patients increased consistently with the increased expression of *CDR2L* and *FSTL3* (Fig. 3E,F). Interestingly, we discovered that a decrease in *FABP4* expression was associated with an increase in *HbA1c* levels. It is likely that this occurs through an indirect mechanism, where the reduction in *FABP4* expression affects lipid metabolism, subsequently leading to an elevation in *HbA1c* levels^39^ (Fig. 3G) (Supplementary Fig. S1).

### GSEA and proteomics analysis

**Fig. 4:**
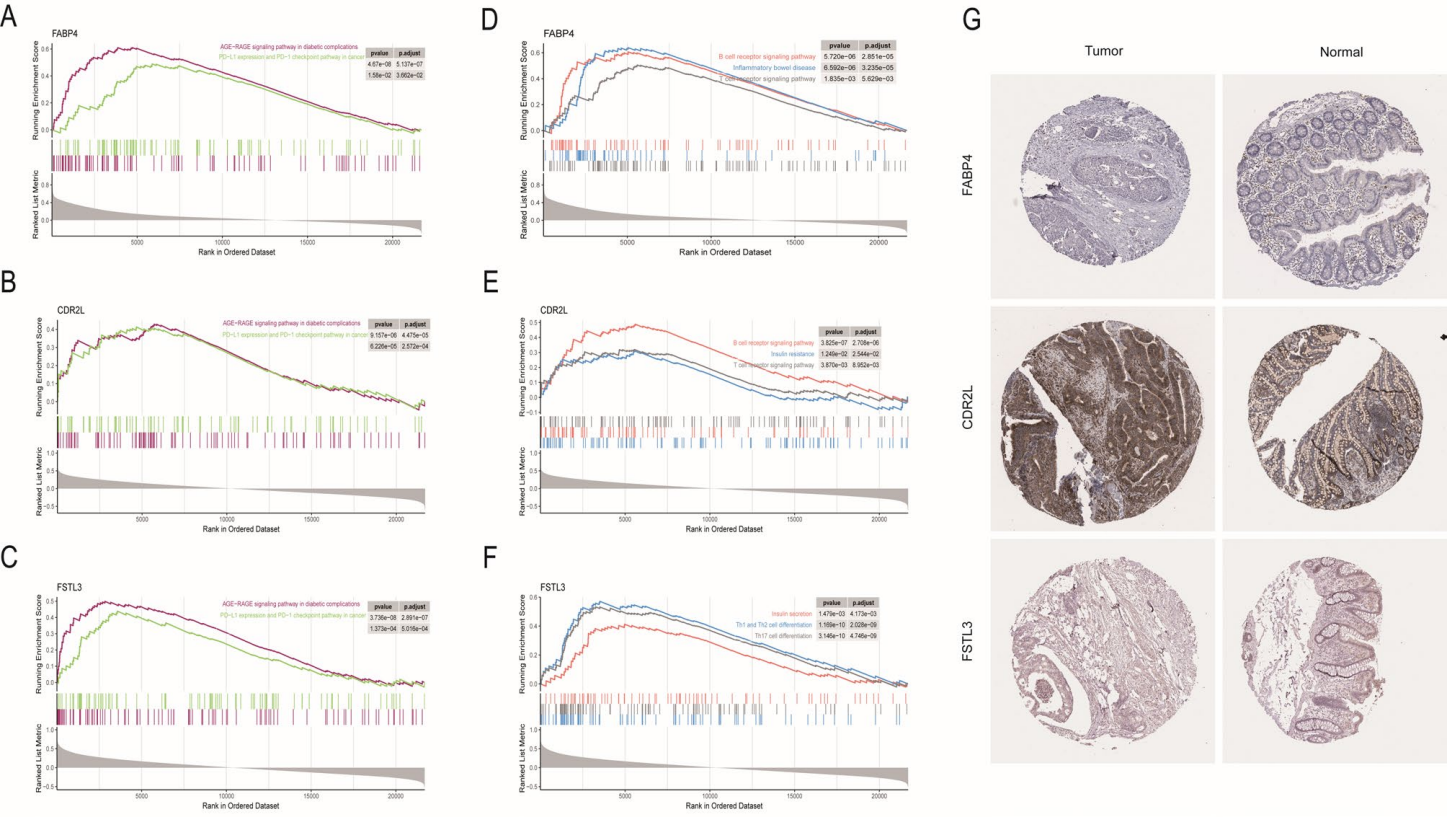
Key gene related proteomics and transcriptomics. (**A–C**) GSEA plots demonstrating significant enrichment of pathways in key gene CRC tumors and T2DM. (**D–F**) GSEA plot showed significant enrichment of key genes in immune pathways. (**G**) The immunohistochemical staining results revealed significant differences of key molecules at the protein expression between normal and tumor tissues.

To further explore the molecular mechanisms underlying the survival outcomes, used the GSEA package to conduct biological process and pathway analyses on the key genes in the TCGA dataset. The results indicated that in cases with high expression of *FABP4*, *CDR2L*, and *FSTL3*, immune-related pathways associated with diabetes and colon cancer were significantly enriched. These pathways included the expression of PD-L1 in cancer and the PD-1 checkpoint pathway, AGE-RAGE signaling in diabetic complications, insulin secretion, insulin resistance, and the differentiation of T cells, B cells, and other cell types (Fig. 4A–F). GSEA was performed using the KEGG dataset with default reference settings, and a *P* < 0.05 was considered to indicate significant enrichment.By validating the immunohistochemical results of the three key molecules in the HPA database, we could observe the immunohistochemical profiles of the proteins in normal and intestinal cancer tissues. There were differences in their protein-level expression (Fig. 4G).

### Risk model prediction

**Fig. 5:**
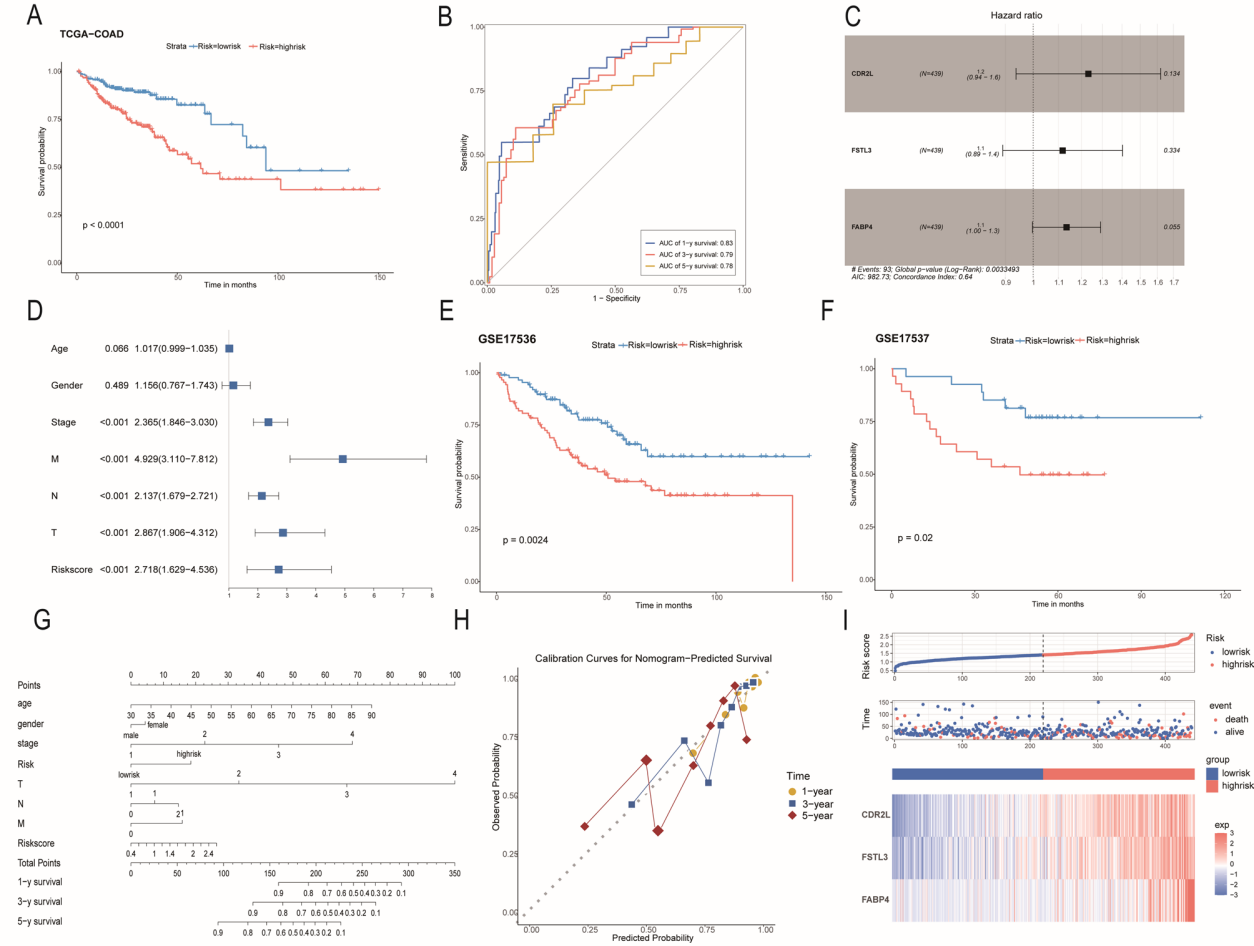
Assessment of the risk model. (**A**) Survival analyses for low (219 samples) and high (220 samples) riskScore groups using Kaplan-Meier curves; *P* < 0.001, Log-rank test); (**B**) 1-, 3- and 5-year OS ROC curves of the key gene signature in the TCGA cohort; (**C, D**) Forest map of (**C**) univariate Cox and (**D**) multivariate Cox regression analysis on the ability of clinical parameters to predict OS in TCGA cohort; (**E,F**) KM curve for validation of the prognosis signature in the(**E**)GSE15736 (**F**)GSE15737 cohorts. (Log-rank test); (**G,H**) (**G**)Nomogram predicting the 1-, 3- and 5-year OS and clinical parameters for CRC patients. (**H**)Calibration plots for 1-, 3- and 5-year OS predictions; (**I**)Risk score distribution in the TCGA cohorts.

CRC genomic profiling data were utilized to construct risk profiles, which were then evaluated using clinical data. A model was developed through stepwise regression to calculate a risk score for each individual. This calculation was based on the variable data from the TCGA-COAD dataset and followed a specific risk formula. The risk score signature was defined as: RiskScore = 0.126*Exp(*FABP4*) + 0.209*Exp(CDR2L) + 0.112*Exp(*FSTL3*). The coefficients were derived from stepwise regression, and “Exp” represented the expression level of key genes. The cohort was divided into low-risk and high-risk groups according to the median risk value of the cohort., the results indicated that patients in the low-risk group had a more favorable survival outcome compared to those in the high-risk group (Fig. 5A).Time-dependent ROC curves were employed to assess the prognostic predictive efficiency of the relevant risk profile. The area under the curve (AUC) values for 1-, 3-, and 5-year overall survival (OS) in the COAD dataset were 0.83, 0.79, and 0.78, respectively(Fig. 5B).Characteristics such as age, gender, tumor stage, grading, and risk scores were selected through multivariate Cox regression analysis. Risk ratios (HR) were quantified with 95% confidence intervals (CI) (Fig. 5C,D). The findings were validated in the GEO dataset cohorts (GSE15737 and GSE15736) (Fig. 5E,F). Column line plots were used to predict the overall survival (OS) of CRC patients. Nomogram demonstrated that the column line plots accurately predicted the 1-, 3-, and 5-year survival times in colon cancer. The plots showed a close correlation with the ideal model and indicated satisfactory agreement (Fig. 5G,H). The outcome data illustrated that the above metrics could serve as independent predictors of prognosis for CRC patients. The scatter plot presented the distribution of genetic risk factors across different samples. In the scatter plot with survival time on the vertical axis, a clustering of high-risk-to-death cases was observed. When combined with the heat map, it was evident that from the gene expression perspective, a redder color indicated a higher relative risk intensity. This suggested that the three genes, *FABP4*, *CDR2L*, and *FSTL3*, showed a consistent and synergistic change trend at the onset of the disease (Fig. 5I). In conclusion, *FABP4*, *CDR2L*, and *FSTL3* can be used as prognostic markers for colon cancer.

### Aberrant expression of co-morbid genes predicts altered immune cells in T2DM and CRC patients

**Fig. 6:**
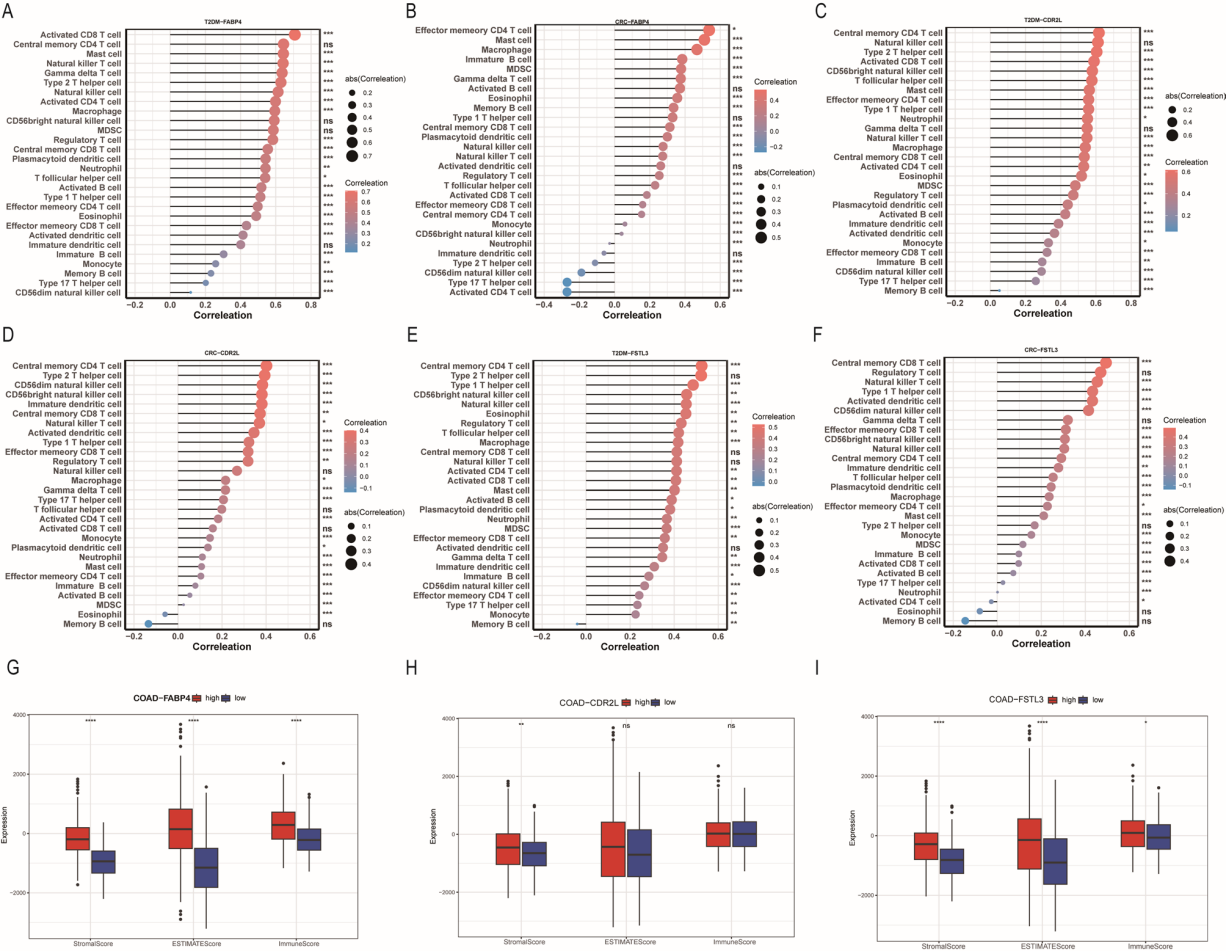
Immune landscape in T2DM and CRC patients with relationship of FABP4, CDR2L, and FSTL3. (**A, B**) Relationship between the expression of FABP4 and immune cell subsets in T2DM (**A**) and CRC (**B**) patients using the ssGSEA algorithm. (**C,D**) Relationship between the expression of CDR2L and immune cell subsets in T2DM (**C**) and CRC (**D**) patients using the ssGSEA algorithm. (**E, F**) Relationship between the expression of FSTL3 and immune cell subsets in T2DM (**E**) and CRC (**F**) patients using the ssGSEA algorithm. (**G-I**) Comparison of Stromalscore, Immunescore, and ESTIMATEScore between high and low expression groups of FABP4 (**G**), CDR2L (**H**), and FSTL3 (**I**). ns: *p *> 0.05, *p < 0.05, **p < 0.01, and ***p < 0.001.

Given the enrichment of key genes in the GSEA pathway, we extrapolated that *FABP4*, *CDR2L*, and *FSTL3* are regulated by immune cells and immune responses. This hypothesis suggests that immune dysfunction associated with *FABP4*, *CDR2*, and *FSTL3* may play a crucial role in the co-morbid development of T2DM and CRC. Ued the ssGSEA algorithm to calculate the abundance of 28 immune cell types in diabetes samples from the T2DM dataset (GSE164416) and tumor samples from the TCGA-COAD cohort. Subsequently, we analyzed the relationship between the expression levels of the three genes and the infiltration levels of each immune cell type in both disease samples (Supplementary Fig. S2).Our findings were as follows: In type 2 diabetes mellitus and colorectal cancer, the following patterns were observed: Firstly, the expression of *FABP4* was negatively correlated with the infiltration of neutrophils, Th2 cells, Th17 cells, CD4 + T cells, and NK cells, while it was positively correlated with the infiltration of the remaining immune cells (Fig. 6A,B). Secondly, the expression of CDR2L was negatively correlated with the infiltration of memory B cells and CD4 + T cells, yet it was positively correlated with the infiltration levels of the vast majority of immune cells (Fig. 6C,D). Thirdly, the expression of FSTL3 was inversely correlated with the degree of infiltration of memory B cells, but positively correlated with the infiltration levels of almost all other immune cells(Fig. 6E,F). Moreover, we used the ESTIMATE algorithm to calculate the StromalScore, ImmuneScore, and ESTIMATEScore of CRC samples in the TCGA-COAD cohort. Then, we compared the differences in these ESTIMATE scores between the low-expression and high-expression samples of the genes. The results indicated that the StromalScore, ImmuneScore, and ESTIMATEScore were significantly higher in the high-expression group of the three key genes than in the low-expression group (*P* < 0.001) (Fig. 6G-I).

### Identification of cell types expressing *FABP4*, *CDR2L*, and *FSTL3* via scRNA-seq analysis

**Fig. 7:**
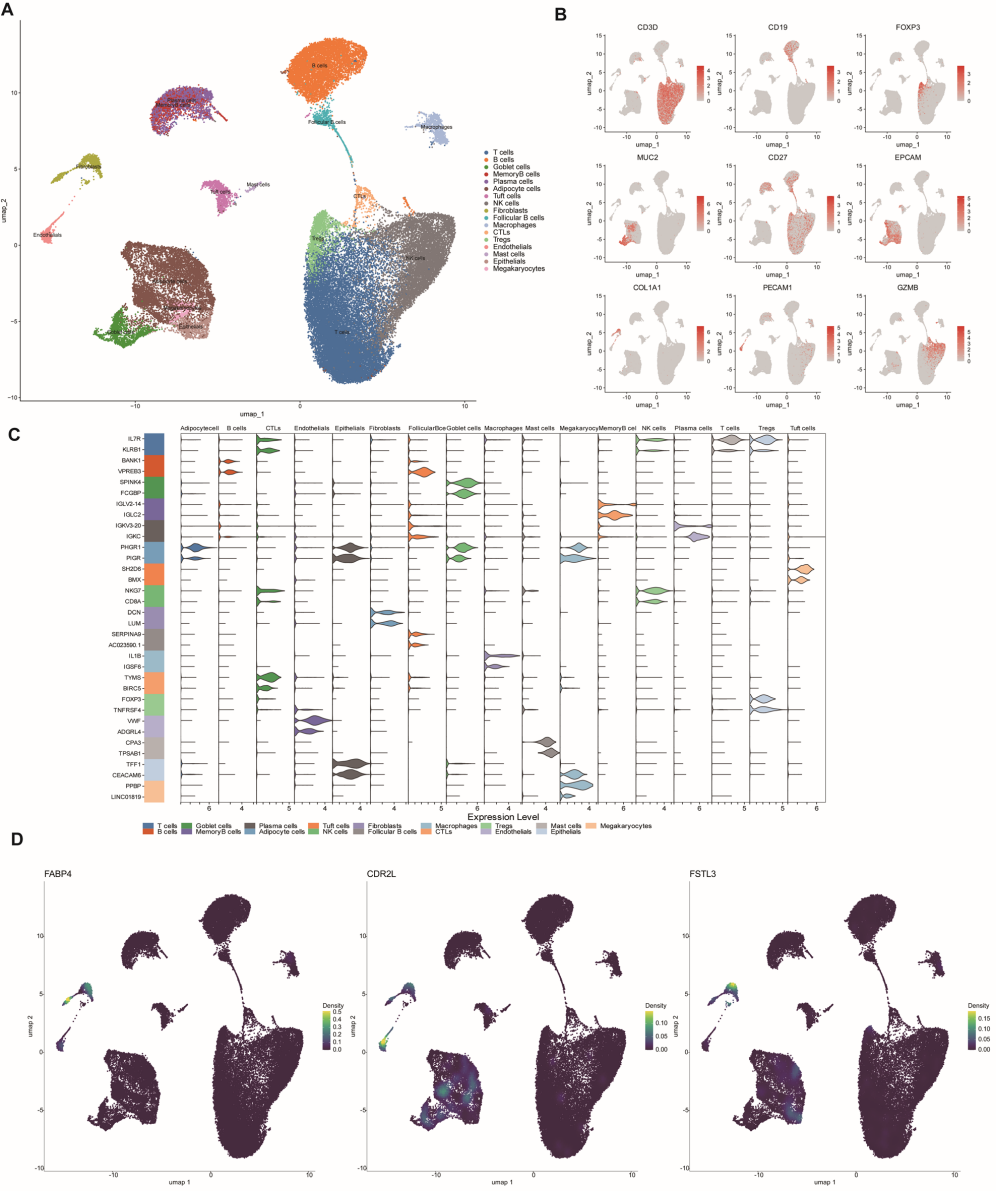
Overview of infiltrating cell types in CRC. (**A**) Umap plot of 49,589 cells from 23 primary CRC samples. (**B**) Violin plot showing the expression of marker genes (Each color represents the expression of a cor responding marker gene in 17 cell subpopulations). (**C**) Umap density plots of the expression of FABP4, CDR2L, and FSTL3 in 17 cell subpopulations. (**D**) Marker gene expression in Single cell sequencing.

To explore the roles of *FABP4*, *CDR2L*, *and FSTL3* in cellular composition and molecular profiles, we analyzed single-cell RNA-sequencing (scRNA-seq) data from 49,589 cells derived from 23 colon cancer samples (GSE200997). After normalizing the data, 3000 variable genes were selected for further analysis (Fig. 7A). Principal component analysis (PCA) was employed to downscale and visualize the data, while UMAP (Uniform Manifold Approximation and Projection) was used to display the specific expression levels of each gene. After integrating the transcriptional data, we first labeled each cell subpopulation based on known cell type-specific markers and then performed manual labeling. As a result, seventeen major cell types were identified: T cells, B cells, Goblet cells, Memory-B cells, Plasma cells, Adipocytes, Tuft cells, NK cells, Fibroblasts, Follicular B cells, Macrophages, CTLs, Tregs, Endothelial cells, Mast cells, Epithelial cells, and Megakaryocytes (Fig. 7B). By using the “FindAllMarkers” function to identify marker genes for different cell subpopulations, the violin plot shows the most representative marker genes for each cell type(Fig. 7C). Additionally, the expression of *CD3D, CD19, FOXP3, MUC2, CD27, EPCAM, COL1A1, PECAM1*, and *GZMB* in the corresponding cell types was presented (Fig. 7D). We observed that *FABP4* was significantly expressed in fibroblasts and endothelial cells, *FSTL3* was significantly expressed in fibroblasts and epithelial cells, and *CDR2L* was significantly expressed in fibroblasts and endothelial cells. Notably, all three key genes showed high expression levels in fibroblasts(Supplementary Fig. S3).

### CAFs alter cytotoxicity of immune cells through cell-to-cell interactions

**Fig. 8:**
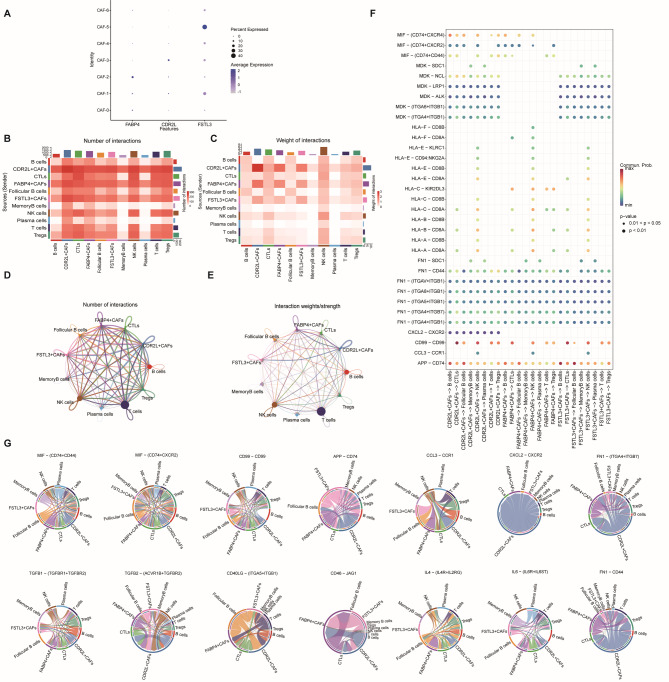
CellChat analysis of the crosstalk between cells in colon cancer. (**A**) Dot plot displaying the distribution of expression levels for key cell type markers. (**B, C**) Comparisons of overall changes in cell-cell communication, including the differential number of interactions (left)(**B**) and differential interaction strength (right) (**C**) between immune cells of rectal cancer compared with colon cancer fibroblasts, with the red representing increased communication. (**D, E**) Heatmaps showing the interaction number (left) (**D**) and interaction strength (right) (**E**) between colon cancer and rectal cancer, with the top color bar representing the sum of the column values displayed in incoming signals and the right color bar representing the sum of outgoing signals. (**F**)Communication probabilities of important ligand-receptor pairs mediated the cell-cell communication from main cell clusters to T cells or B cells. The color of the dot represents the probability of communication, and the size of the dot corresponds to p-value. The ligand receptor corresponding to empty meaning does not mediate communication in this cell. (**G**)chord plots showing major signaling pathways.

To investigate intercellular crosstalk in colon cancer, we employed the CellChat tool to systematically evaluate potential fibroblast-immune cell interactions by analyzing communication frequency and interaction weights. Cancer-associated fibroblasts (CAFs), the predominant stromal component in tumor microenvironments, promote oncogenesis through multiple mechanisms. These cells modulate critical tumor cell behaviors including proliferation, invasion, migration, and angiogenesis via secretion of cytokines, growth factors, and extracellular matrix components. Through cluster analysis of 871 fibroblasts (Supplementary Fig. S4), we identified seven distinct subsets. Using bubble plot visualization and marker annotation (Fig. 8A), three subpopulations-*FABP4 + CAFs*, *CDR2L + CAFs*, and *FSTL3 + CAFs*-were characterized. Comparative analysis revealed differential ligand-receptor interaction probabilities between these CAF subsets and immune cell populations in colorectal cancer. Quantitative analysis of signaling flux demonstrated that *FABP4 + CAFs*,* CDR2L + CAFs*, and *FSTL3 + CAFs* exhibited enhanced interaction frequency and strength with T/B lymphocytes compared to normal controls (Fig. 8B–E). Given the pivotal role of fibroblast heterogeneity in colorectal cancer (CRC) microenvironment organization, systematic mapping of intercellular signaling networks becomes crucial for understanding tumor-stromal crosstalk. At the efferent signaling terminus, *FABP4 + CAFs*, *CDR2L + CAFs*, and *FSTL3 + CAFs* function as secretory effectors, primarily utilizing multiple signaling modalities. Key pathways including ligands such as *MIF*,* CD99*, *CCL*, *CXCL*, *CD40*, *CD46*, *IL*, *TGF-β*, *FN* and *APP* exhibited elevated activation during fibroblast-T/B lymphocyte crosstalk (Fig. 8F). The pleiotropic ligand MIF operates as a chemokinetic inflammatory mediator, with its *CD74*-containing receptor complexes coordinating inflammatory cascades. Concurrently, *CD99* -mediated homophilic interactions regulate cellular adhesion and immunomodulatory processes. Chemokines (*CCL*, *CXCL*) facilitated directional immune cell trafficking, while immune checkpoint molecules (*CD40*, *CD46*) modulated response thresholds. Multifaceted cytokine networks (*IL*, *TGF-β*, *FN*) dynamically regulated immune cell homeostasis, collectively establishing an immune niche (Fig. 8G) (Supplementary Fig. S5). Fibroblasts, as an important member of stromal cells, are capable of producing different cytokines. These cytokines may modulate the composition of immune cells, leading to a shift towards a more anti-tumor-friendly structure, which in turn can influence tumor immune surveillance, which is essential for orchestrating a robust anti-tumor immune response.

### Expression of *FABP4*, *CDR2L*, and *FSTL3* predicts the benefit of immunotherapy in COAD

**Fig. 9:**
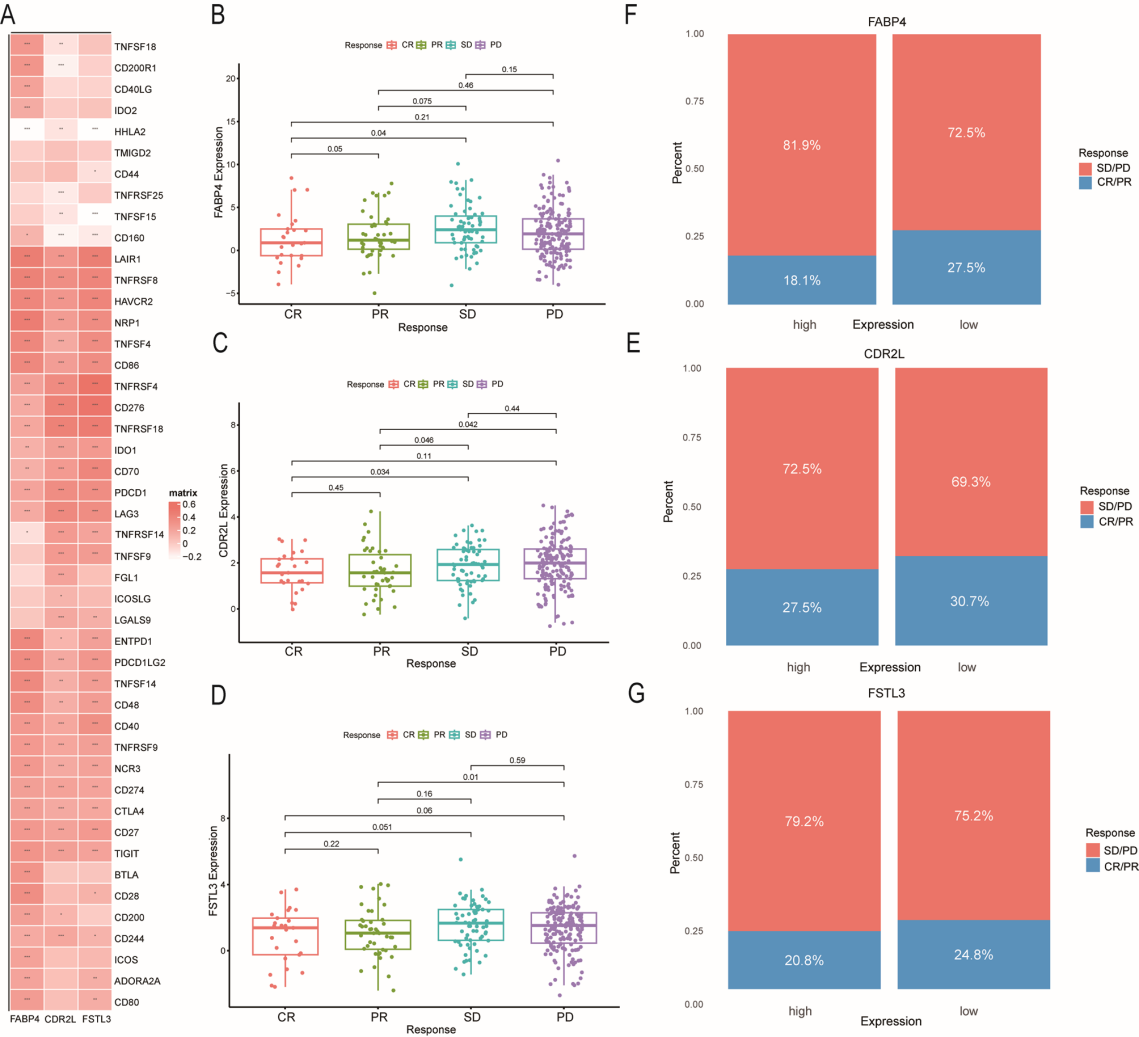
Key gene expression predicts the benefit of immunotherapy. (**A**)Relationship between the key gene expression and immune checkpoints (**B–D**) FABP4, CDR2L, and FSTL3 expression levels across different anti-PD-L1 clinical response groups. (**E–G**) Comparison of immunotherapy outcomes in patients with high or low FABP4, CDR2L, and FSTL3 expression receiving anti-PD-L1 treatment. CR, complete response; PR, partial response; SD, stable disease; PD, progressive disease.

Based on Xu’s study^40^, immune checkpoints, serving as the latest therapeutic targets for colon cancer, play a crucial role in tumor immunotherapy. In the TCGA-COAD dataset, most immune checkpoints were positively correlated with *FABP4*, *CDR2L*, and *FSTL3* (Fig. 9A). Given the critical roles of PD-1 and PD-L1 in tumor immunosuppression and therapy, we investigated the potential impacts of the expression levels of these key genes on the immunotherapy response.The IMvigor210 cohort was selected to analyze the effects of high and low gene expression levels on the response to PD-1 monoclonal antibody immunotherapy. The results demonstrated that patients with low expression levels of these genes exhibited better disease control and higher complete remission rates compared to those with high expression levels. (Fig. 9B–G)

## Discussion

A meta-analysis of 15 studies involving over 2.5 million patients revealed that the relative risk of colorectal cancer was approximately 30% higher in diabetic patients compared to non-diabetic patients^41^. Several biological mechanisms can account for the association between diabetes and the increased risk of CRC. These mechanisms include high blood glucose and high blood lipids resulting from insulin resistance, and active energy metabolism that promotes the growth of CRC tumor cells^42^. Moreover, the high metabolic environment creates conditions for tumorigenesis. It can supply sufficient glucose to meet the demands of tumor cell proliferation, thereby supporting tumor growth and development. Insulin-like growth factor (*IGF*) shares structural and functional similarities with insulin, and they have a common part in the signaling pathway^43^. High levels of *IGF-1* can disrupt insulin signaling by competing for insulin receptor substrate (IRS). This disruption impairs insulin’s function in promoting glucose uptake and glycogen synthesis, thus worsening insulin resistance^44,45^. On the other hand, *IGF* enhances the anti-apoptotic, migratory, and invasive capabilities of colon cancer cells^46,47^. The genes identified in this study happen to be tightly linked to hypermetabolism and IGF, elucidating a common mechanism between T2DM and CRC.

Fatty acid binding proteins (*FABPs*) are a class of highly conserved small cytoplasmic proteins that bind long-chain fatty acids and other hydrophobic ligands. Our study shows that *FABP4*, one of these proteins, is highly expressed in adipose tissues and macrophages. *FABP4* is extensively involved in the regulation of glucose and lipid metabolism pathways related to inflammation and metabolic processes. It influences the insulin signaling pathway by modulating fatty acid uptake and metabolism, which in turn affects insulin sensitivity and increases insulin resistance^48^. It has been found that higher lipid accumulation and stronger *FABP4* transcripts were observed in colon cancer tissues. When incubated with adipose tissue extracts and overexpressed *FABP4*, colon cancer cells exhibited enhanced lipid accumulation and significantly enhanced invasion and migration^49^. In *FABP4*-overexpressing colon cancer cells, the expression of the *AKT* pathway and epithelial-mesenchymal transition (*EMT*)-related proteins was regulated. The activation of these signaling pathways was closely associated with colon cancer invasion and metastasis^50^. The follicle suppressor modular protein family (*FST*), a secreted protein, is widely present in and secreted by various tissues and organs in mammals and plays a crucial role in hormone regulation, energy metabolism, and muscle and adipose tissue proliferation and differentiation^51^. Folliculostatin-like 3 *(FSTL3*), a member of the folliculostatin modular protein family, can participate in signaling involving members of the *TGFB* family and in the regulation of the insulin-like growth factor (*IGF*) transporter and uptake by the insulin-like growth factor-binding protein (*IGFBP*)^52,53^. It can also contribute to colorectal cell growth through the Wnt/β-catenin-mediated epithelial-mesenchymal transition (*EMT*) and aerobic glycolysis, promoting colon cancer invasion and metastasis. High expression of *FSTL3* is associated with lymph node metastasis and can serve as a biomarker for extracellular matrix remodeling in colon cancer^54,55^. Cerebellar degeneration-associated protein 2 (*CDR2L*), a key focus in neurological-related research, is involved in complex processes such as inter-neuronal signaling and has a specific chromosomal localization region. It is associated with some neurodegenerative diseases, such as Alzheimer’s and Parkinson’s^56^. *CDR2L* is widely present in ovarian cancer tissues and abundantly expressed in testicular and prostate cancer tissues. It can inhibit the function of the oncogene (*c-MYC*), a major regulator of cell growth and cellular metabolism, through its cytoplasmic segregation^57,58^. *CDR2L* is also involved in signaling and protein interactions for gene transcription, such as those with cell cycle-associated proteins and in activated serine/threonine protein kinase pathways^59–61^. Relatively few studies have been conducted on this gene in diabetes and colon cancer. The current study, along with *FABP4* and *FSTL3*, explored its potential function and mechanism in T2DM and CRC diseases.

This study aimed to validate the roles of co-expressed genes in the diagnosis, treatment selection, and prognosis of colorectal cancer and type 2 diabetes mellitus by utilizing multiple datasets. CRC patients were classified into high-risk and low-risk groups according to their risk scores. The clinical overall survival (OS) outcomes of the high-risk group were significantly worse. Multifactorial Cox regression analysis, along with a nomogram showing the agreement between 1-year, 3-year, and 5-year OS prediction and observation rates, indicated that the gene models of *FABP4*, *CDR2L*, and *FSTL3* were independent risk factors for OS. Subsequently, a comparative assessment of the immunologic profiles of patients with high and low expression levels of the aforementioned genes was carried out using the Single-Sample Gene Set Enrichment Analysis (SSGSEA) and ESTIMATE algorithms. The ESTIMATE data revealed that the immune score, stromal score, and ESTIMATE score were higher in the high-expression group compared to the low-expression group. This suggests that the tumor immune microenvironment was more active in the high-expression group. Notably, the degree of immune cell infiltration into tumors has been associated with tumor growth, progression, and prognosis, making these aspects crucial areas of recent research^62^. SSGSEA is a gene set enrichment analysis method, mainly used to evaluate the degree of gene set enrichment in a single sample. By analyzing the expression levels of the obtained genes in 28 immune cell subtypes, including but not limited to natural killer T cells, CD4 + T cells, Th1 cells, Th2 cells, Th17 cells, and B cells, significant enrichment was observed in these subtypes. It has been found that individuals with upregulated levels of helper T cells tend to exhibit excessive inflammatory responses and a poorer prognostic status^63^. It’s worth looking at as antigen-presenting cells, B cells can effectively activate T cells in response to PD-1 blockade. This activation, in turn, strongly promotes the anti-tumor immune process^64^. Moreover, NK cells have the ability to induce immunotoxicity in tumor cells. Therefore, when the infiltration level of NK cells increases, the prognosis of CRC patients is likely to improve^65^. In addition, during an in-depth study of PD-1-resistant tumor models, researchers were astonished to find that NK cells have a remarkable ability to reactivate exhausted CD8 + T cells^66^. From our current series of studies on the immune architecture, it is evident that in the CRC patient population, the degree of immune cell infiltration is significantly higher in the group with high expression of key genes compared to the group with low expression. Based on this, it is reasonable to hypothesize that the high-expression group may possess a more robust immune capacity. As a result, this group may be a more suitable candidate for immunotherapy and is expected to achieve more favorable treatment outcomes in subsequent therapies.

Over the years, cancer-associated fibroblasts (CAFs) have been thoroughly characterized by their multiple properties in promoting cancer development and maintaining the homeostasis of the tumor microenvironment. This makes them a promising key target for anticancer therapy and has attracted numerous clinical trials that focus on CAFs and their associated signaling pathways. Owing to the rapid advancement of single-cell sequencing technology, we have been able to explore in depth the ecosystem of the tumor microenvironment (TME) and perform gene expression profiling on individual CAF cells. Previous research has indicated that CAFs are closely related to the secretion of a variety of cytokines, such as chemokines (*CXCL*), interleukins (*IL-1*, *IL-2*, *IL-6*), and tumor necrosis factors (*TNF-β*). These cytokines not only significantly disrupt insulin signaling pathways and exacerbate insulin resistance^67–70^, but also have the function of altering the activity of immune cells. The information flow mediates the intercellular crosstalk between CAFs and T/B lymphocytes, thus strengthening the immunosuppressive microenvironment of CRC. Although we were able to identify various cytokines as ligands that interact with fibroblasts with high expression of key genes, we were unable to confirm whether *FABP4*, *CDR2L*, and *FSTL3* directly participate in intercellular crosstalk as receptors of T/B lymphocytes. It is most likely that they predict the activation of the body’s reactive antitumor immunity in response to another stimulus.

The success of immunotherapy is increasingly recognized as being partly due to the immune landscape of the tumor microenvironment^71^. The PD-1/PD-L1 immunotherapy based on bladder cancer further demonstrated that high levels of *FABP4*, *CDR2L*, and *FSTL3* expression were associated with a poor response to immunotherapy, confirming the applicability of the associated immune dysregulation across various tissues and diseases.

This study conducted a comprehensive analysis of gene expression and immune microenvironment heterogeneity in colorectal cancer (CRC) and type 2 diabetes mellitus (T2DM), aiming to evaluate their diagnostic and prognostic significance and investigate immune cell infiltration levels through pathway enrichment analysis of individual genes. Furthermore, cellular communication networks and transcription factors associated with fibroblast-specific genes were characterized through integration of single-cell RNA sequencing (scRNA-seq) data. These findings may contribute to the development of targeted therapeutic strategies for patients with T2DM and CRC.

## Electronic supplementary material

Below is the link to the electronic supplementary material.

Supplementary Material 1

Supplementary Material 2

Supplementary Material 3

Supplementary Material 4

Supplementary Material 5

Supplementary Material 6

Supplementary Material 7

Supplementary Material 8

Supplementary Material 9

Supplementary Material 10

Supplementary Material 11

Supplementary Material 12

Supplementary Material 13

Supplementary Material 14

## Data Availability

This study was based on publicly available deidentified data.MSigDB database data have been deposited at (https://www.gsea-msigdb.org/gsea/msigdb).The Human Protein Atlas (HPA) data have been deposited at (https://www.proteinatlas.org/)Imvigor210 database have been deposited at (http://research-pub.Gene.com/imvigor210corebiologies). Gene Expression Omnibus (GEO) database have been deposited at (https://www.ncbi.nlm.nih.gov/geo). Cancer Genome Atlas (TCGA) database have been deposited at (https://portal.gdc.cancer.gov). GSE118139 database have been deposited at (https://www.ncbi.nlm.nih.gov/geo/query/acc.cgi?acc=GSE118139). GSE164416 database have been deposited at (https://www.ncbi.nlm.nih.gov/geo/query/acc.cgi?acc=GSE164416). GSE184050 database have been deposited at (https://www.ncbi.nlm.nih.gov/geo/query/acc.cgi?acc=GSE184050). GSE25724 database have been deposited at (https://www.ncbi.nlm.nih.gov/geo/query/acc.cgi?acc=GSE25724). GSE38642 database have been deposited at (https://www.ncbi.nlm.nih.gov/geo/query/acc.cgi?acc=GSE38642). GSE50397 database have been deposited at (https://www.ncbi.nlm.nih.gov/geo/query/acc.cgi?acc=GSE50397). GSE50244 database have been deposited at (https://www.ncbi.nlm.nih.gov/geo/query/acc.cgi?acc=GSE50244). GSE9348 database have been deposited at (https://www.ncbi.nlm.nih.gov/geo/query/acc.cgi?acc=GSE9348). GSE113513 database have been deposited at (https://www.ncbi.nlm.nih.gov/geo/query/acc.cgi?acc=GSE113513). GSE17536 database have been deposited at (https://www.ncbi.nlm.nih.gov/geo/query/acc.cgi?acc=GSE17536). GSE17537 database have been deposited at (https://www.ncbi.nlm.nih.gov/geo/query/acc.cgi?acc=GSE17537). GSE200997 database have been deposited at (https://www.ncbi.nlm.nih.gov/geo/query/acc.cgi?acc=GSE200997).

## Abbreviations

T2DM: Type 2 diabetes mellitus
CRC: Colorectal cancer
DEGs: Differentially expressed genes
TCGA: The cancer genome atlas
GEO: Gene expression omnibus
OS: Overall survival
CPM: Counts per million
scRNA-seq: Single-cell RNA sequencing
PPI: Protein–protein interaction
KEGG: Kyoto Encyclopedia of Genes and Genomes
GO: Gene ontology
ssGSEA: Single-sample gene-set enrichment analysis
GSEA: Gene-set enrichment analysis
HPA: Human protein atlas
DT: Decision tree
LASSO: Least absolute shrinkage and selection operator
GBM: Gradient boosting machine
RF: Random forest
GLM: Generalized linear model
kNN: K-nearest neighbors
NNET: Neural network
SVM: Support vector machine
PCA: Principal component analysis
UMAP: Uniform manifold approximation and projection
