# Supplementary material for: Integrated analysis of shared gene expression signatures and immune microenvironment heterogeneity in type 2 diabetes mellitus and colorectal cancer
Source: Sci Rep. 2025 Jul 1;15:22234. doi: 10.1038/s41598-025-07015-4 (PMC12215021; doi:10.1038/s41598-025-07015-4)
Supplement: Supplementary file 1 — Supplementary Material 1 [file 41598_2025_7015_MOESM1_ESM.docx]

**Supporting information**

**Table S1**:The details and download sources of T2DM and CRC datasets for the research

**Table S2**: Results of log-rank analysis of the initial screened DEG

**Table S3**: Results of Cox analysis

**Supporting Fig. S1**: Scatterplot of FABP4, CDR2L and FSTL3 expression and HbA1c level in the GSE50244 and GSE50397 dataset.

**Supporting Fig. S2**: Immune infiltration levels in high-gene and low-gene groups analyzed by CIBERSORT. * *p* < 0.05, ** *p* < 0.01, *** *p* < 0.001 and ns > 0.05

**Supporting Fig. S3**: Box plot comparing FABP4 (**A**), CDR2L (**B**) and FSTL3(**C**).expression levels across different cell subclusters.

**Supporting Fig. S4**: (**A**) Umap plot of 871 CAFs from CRC samples. (**B**) Umap plot of representative genes of fibroblasts.

**Supporting Fig. S5**: Profile of incoming and outgoing information flows mediated by different signaling pathways in the cell clusters.
