# Supplementary figures and images for: Integrated analysis of shared gene expression signatures and immune microenvironment heterogeneity in type 2 diabetes mellitus and colorectal cancer
Source: Sci Rep. 2025 Jul 1;15:22234. doi: 10.1038/s41598-025-07015-4 (PMC12215021; doi:10.1038/s41598-025-07015-4)

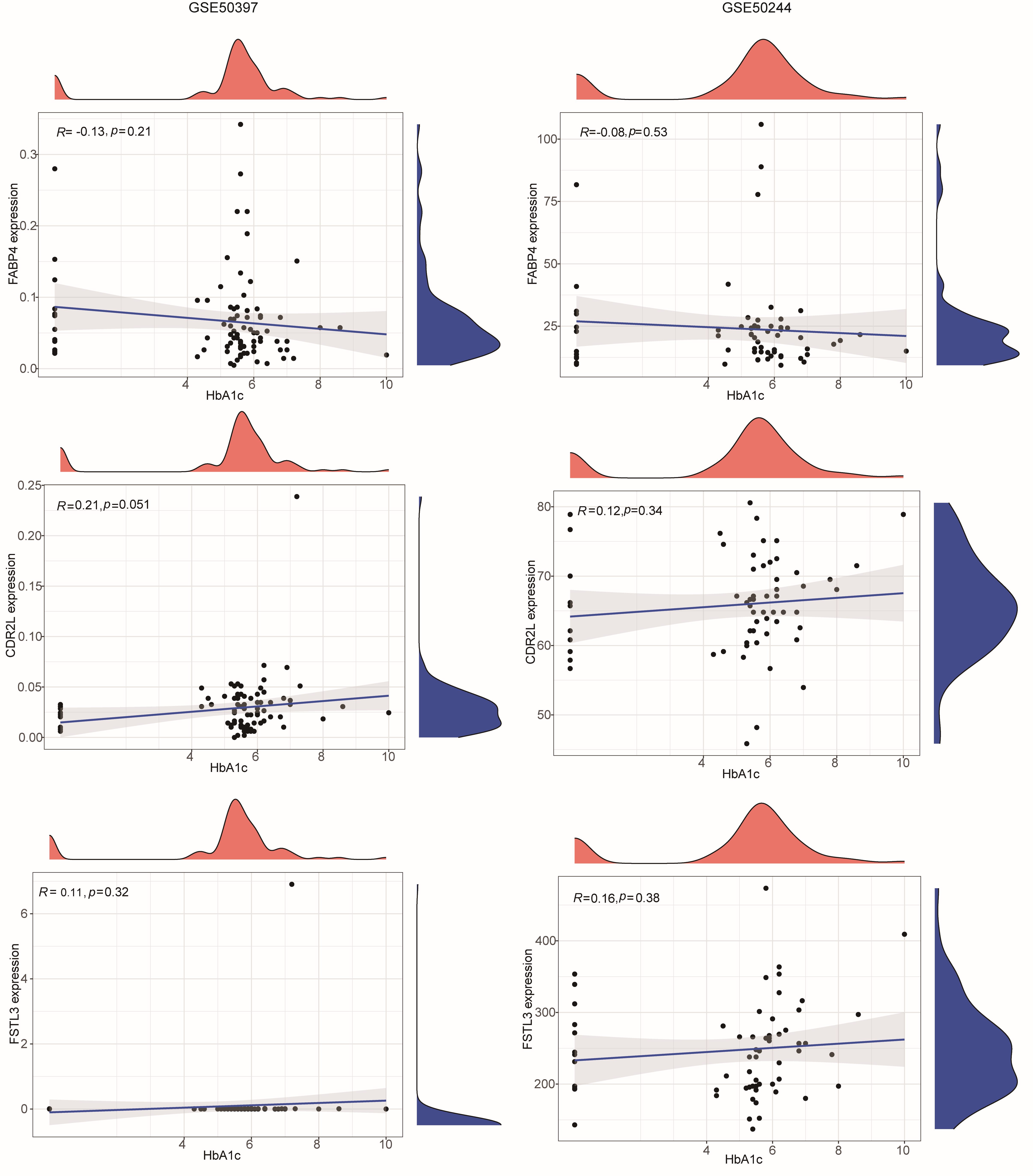

Supplement: Supplementary file 5 — Supplementary Material 5 [file 41598_2025_7015_MOESM5_ESM.tif]

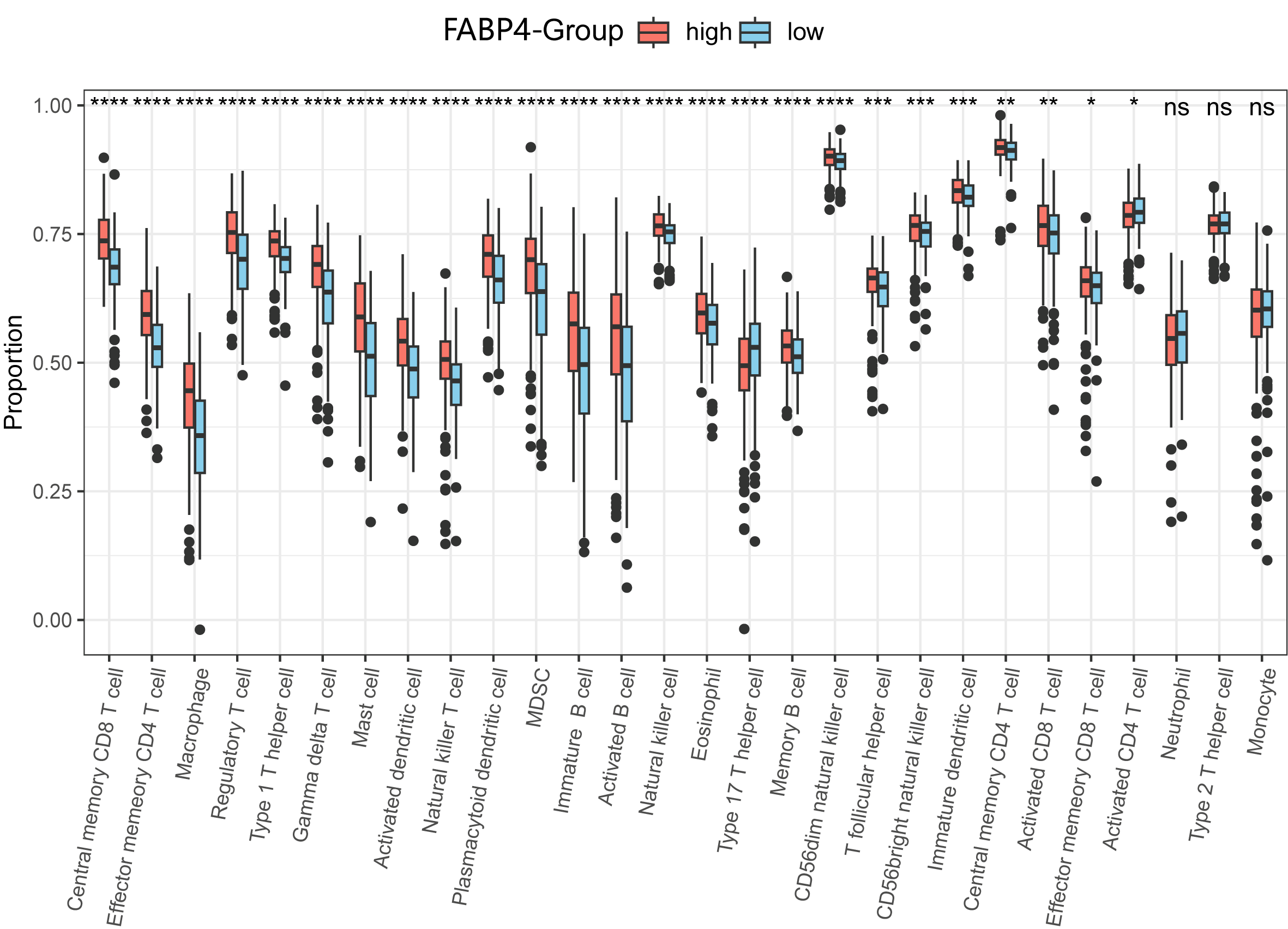

Supplement: Supplementary file 6 — Supplementary Material 6 [file 41598_2025_7015_MOESM6_ESM.tif]

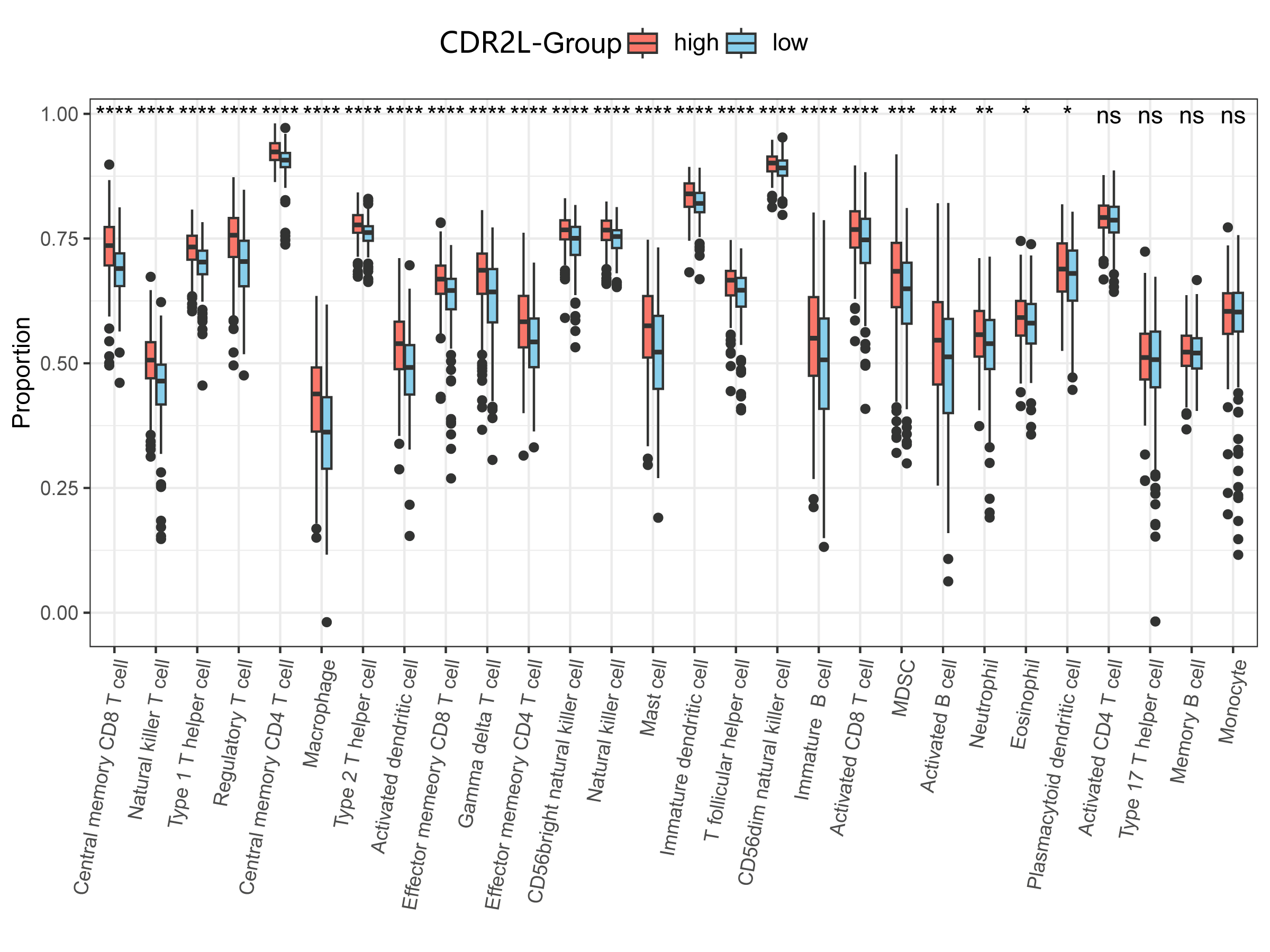

Supplement: Supplementary file 7 — Supplementary Material 7 [file 41598_2025_7015_MOESM7_ESM.tif]

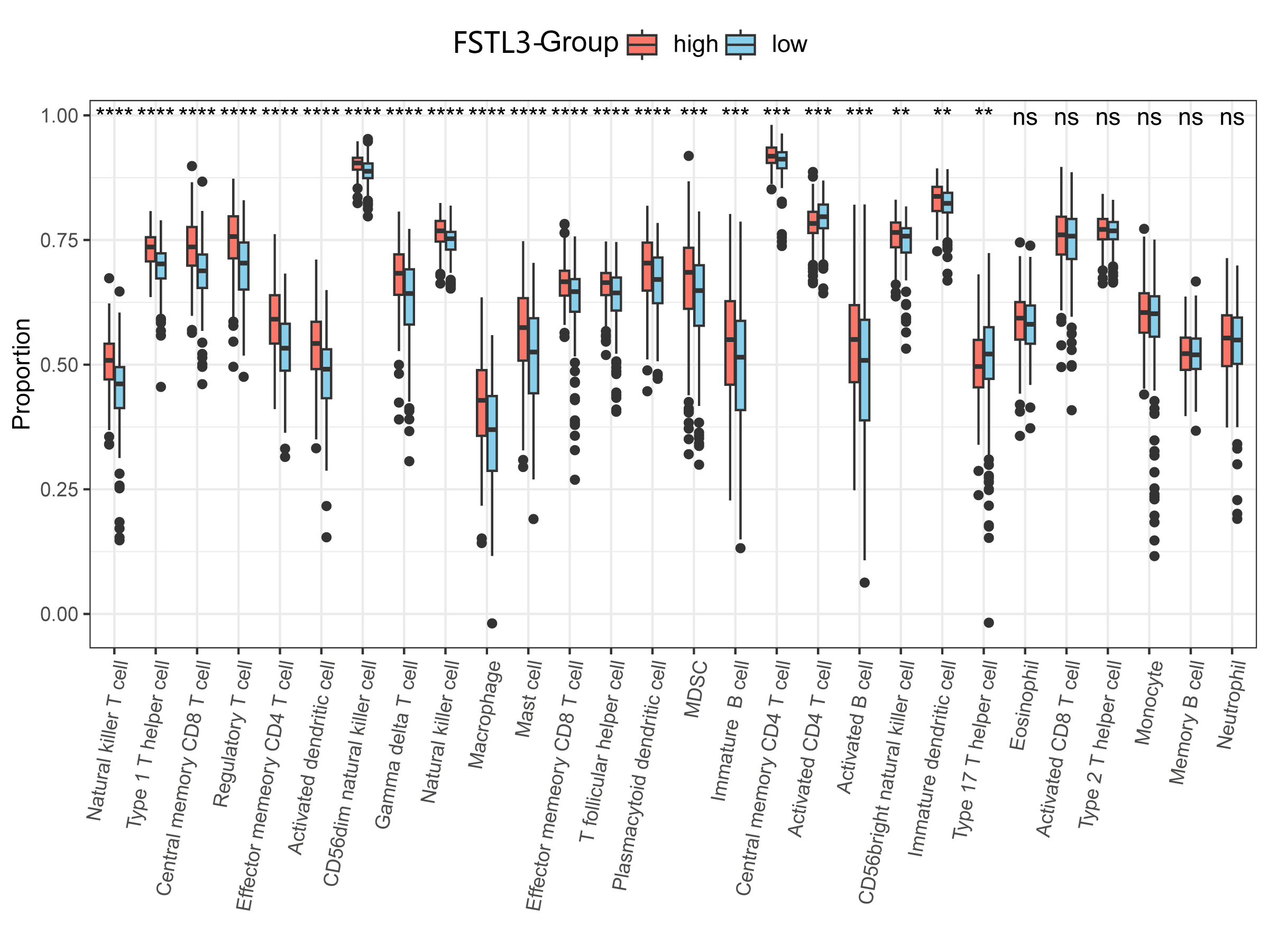

Supplement: Supplementary file 8 — Supplementary Material 8 [file 41598_2025_7015_MOESM8_ESM.tif]

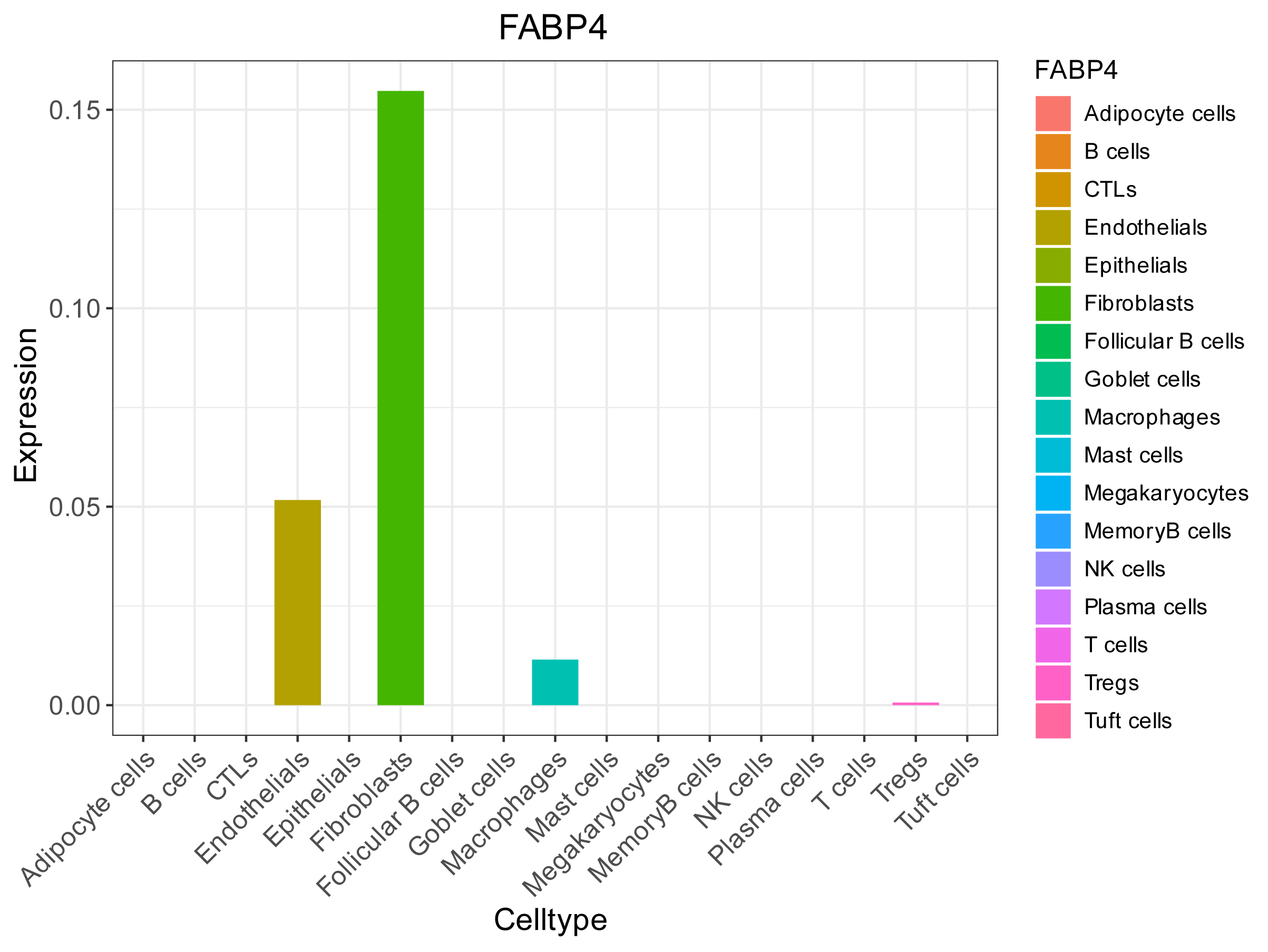

Supplement: Supplementary file 9 — Supplementary Material 9 [file 41598_2025_7015_MOESM9_ESM.tif]

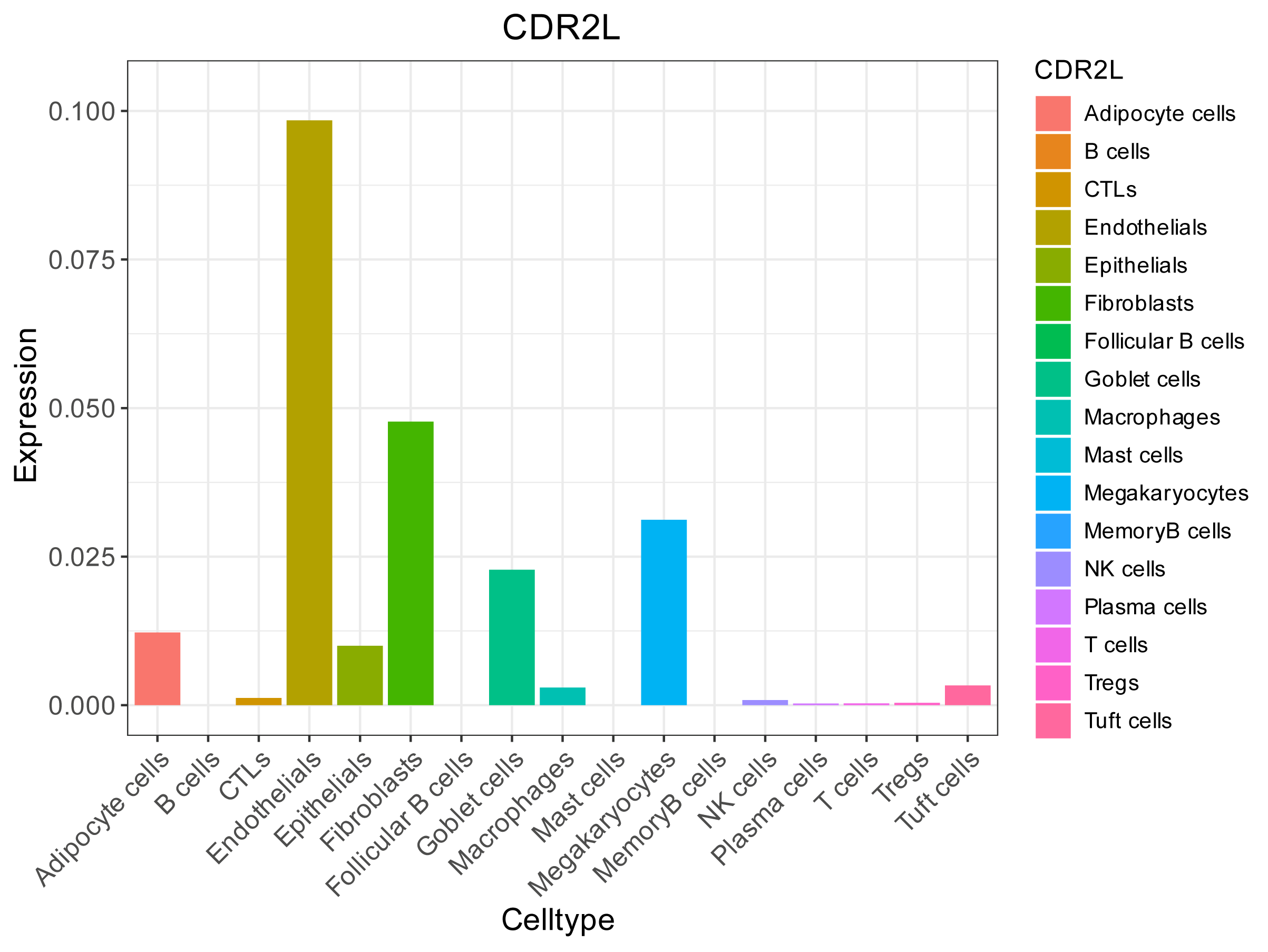

Supplement: Supplementary file 10 — Supplementary Material 10 [file 41598_2025_7015_MOESM10_ESM.tif]

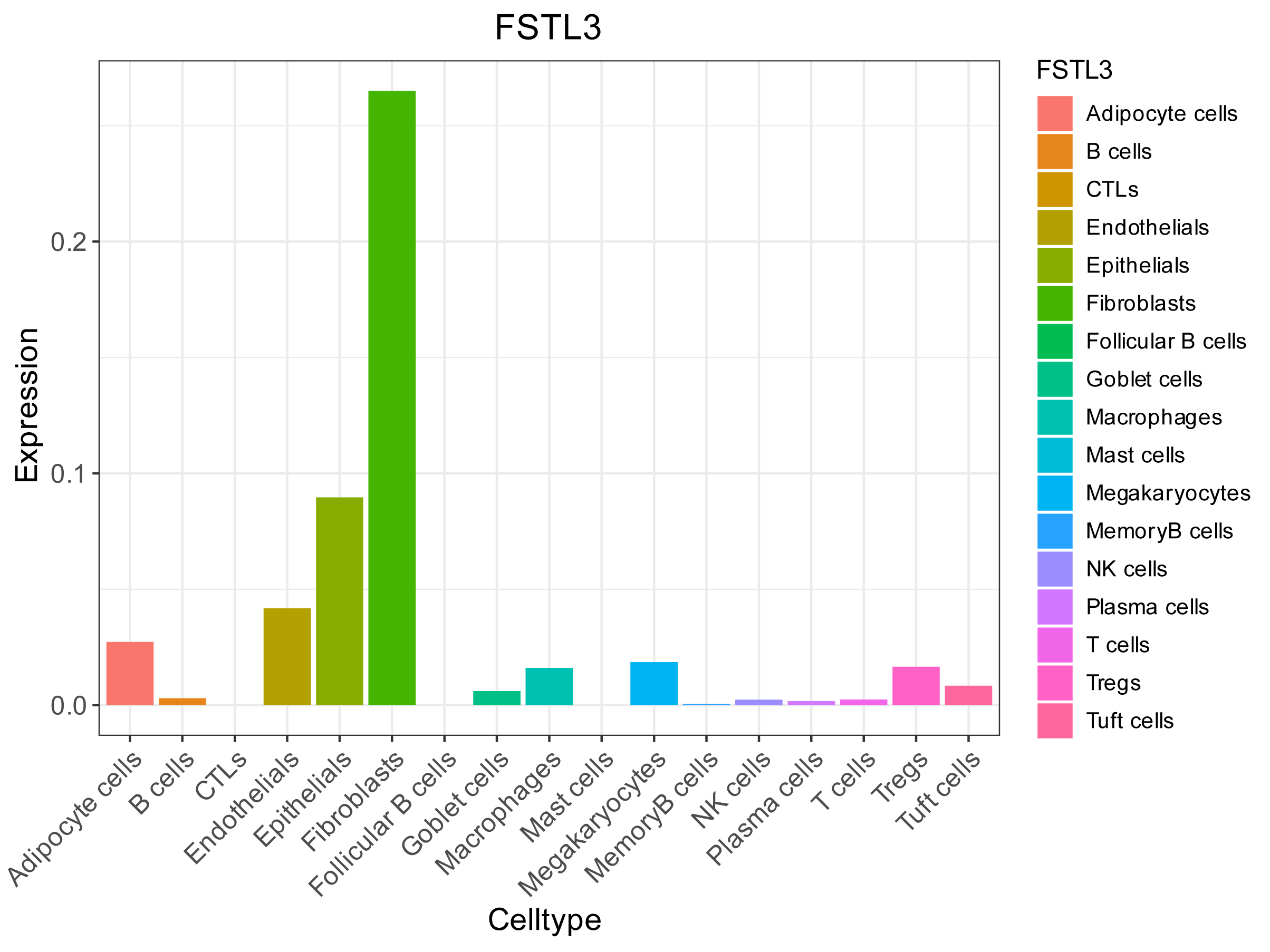

Supplement: Supplementary file 11 — Supplementary Material 11 [file 41598_2025_7015_MOESM11_ESM.tif]

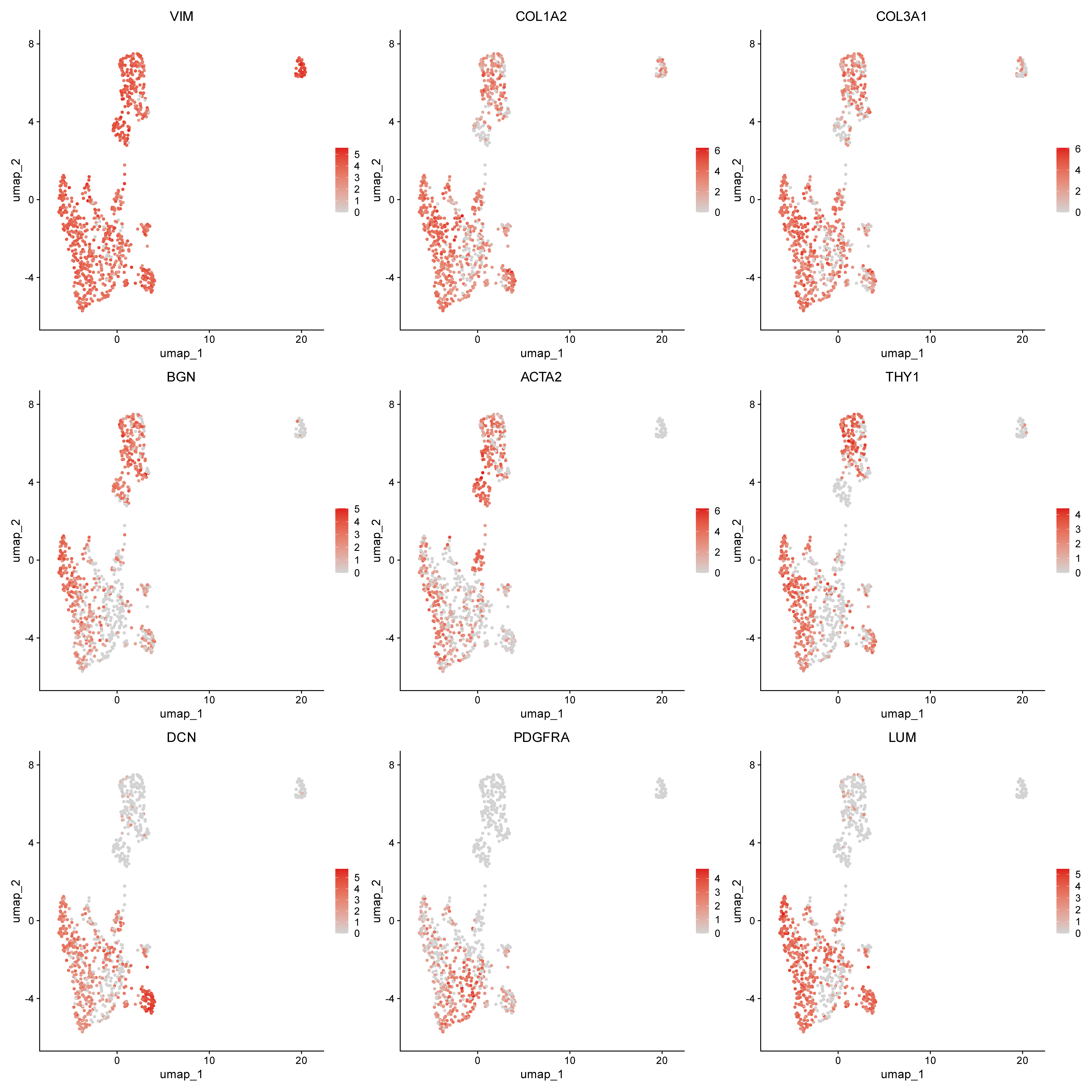

Supplement: Supplementary file 12 — Supplementary Material 12 [file 41598_2025_7015_MOESM12_ESM.tif]

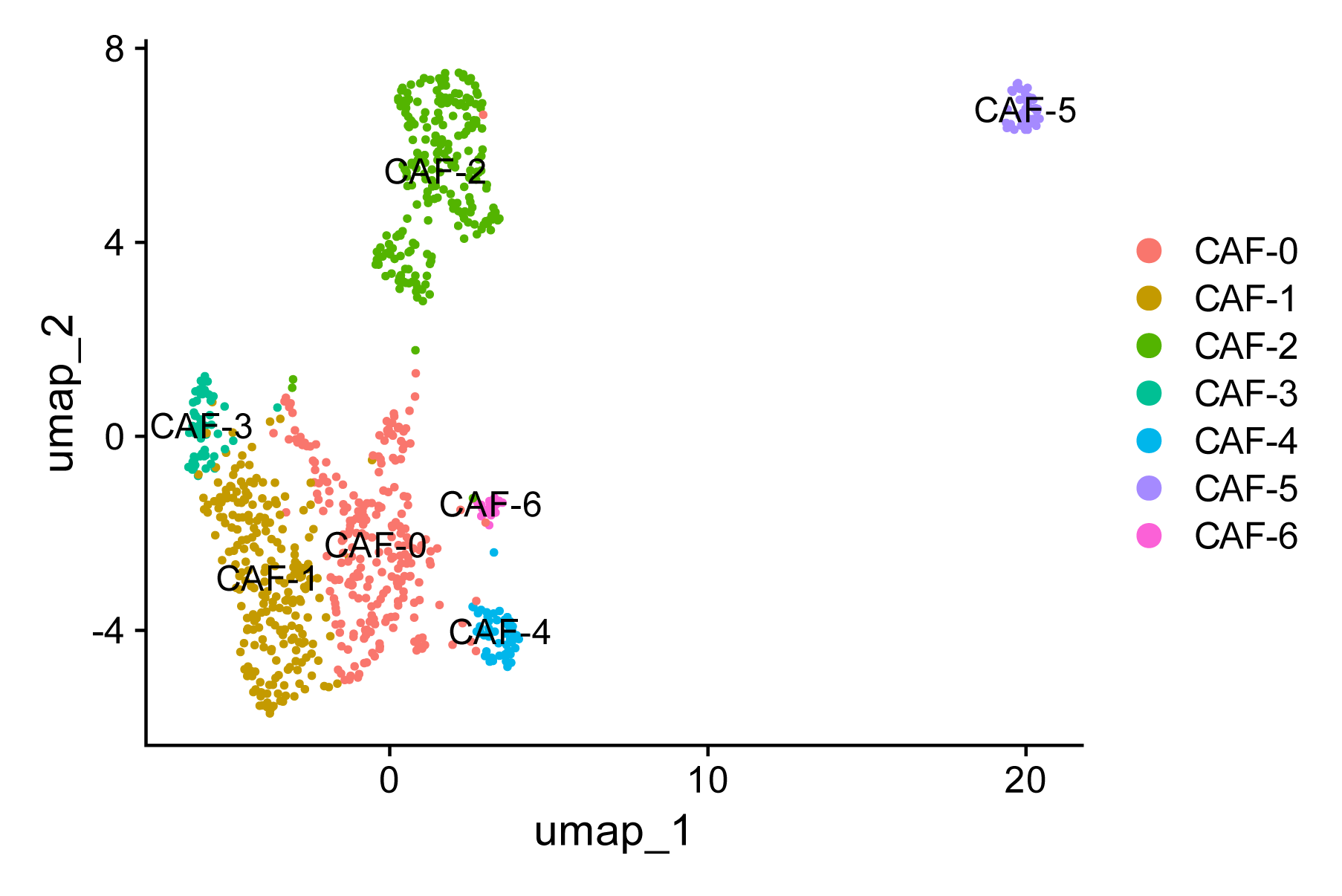

Supplement: Supplementary file 13 — Supplementary Material 13 [file 41598_2025_7015_MOESM13_ESM.tif]

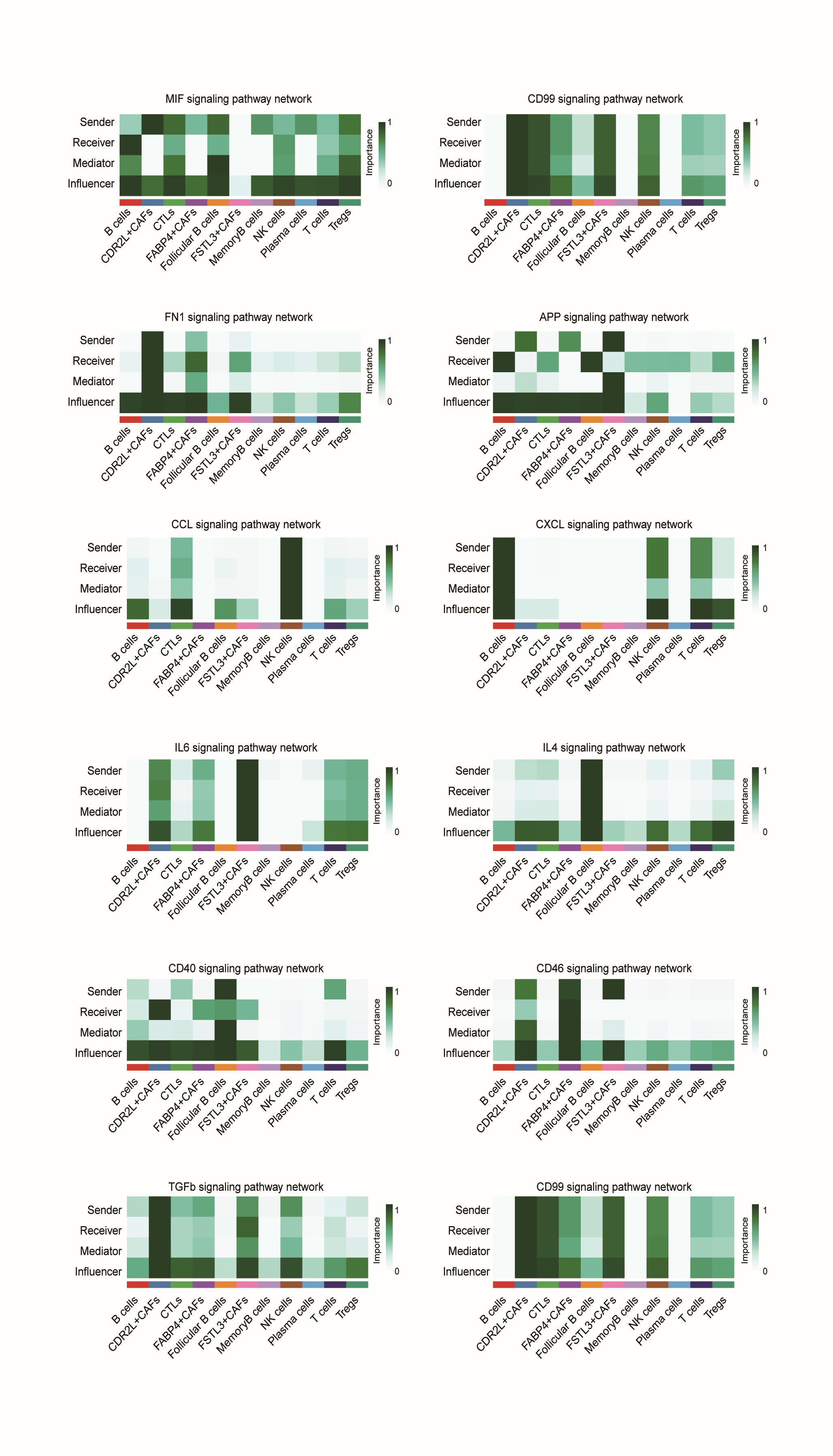

Supplement: Supplementary file 14 — Supplementary Material 14 [file 41598_2025_7015_MOESM14_ESM.tif]
